# Data-driven computational models of ventricular-arterial hemodynamics in pediatric pulmonary arterial hypertension

**DOI:** 10.3389/fphys.2022.958734

**Published:** 2022-09-07

**Authors:** Christopher Tossas-Betancourt, Nathan Y. Li, Sheikh M. Shavik, Katherine Afton, Brian Beckman, Wendy Whiteside, Mary K. Olive, Heang M. Lim, Jimmy C. Lu, Christina M. Phelps, Robert J. Gajarski, Simon Lee, David A. Nordsletten, Ronald G. Grifka, Adam L. Dorfman, Seungik Baek, Lik Chuan Lee, C. Alberto Figueroa

**Affiliations:** ^1^ Department of Biomedical Engineering, University of Michigan, Ann Arbor, MI, United States; ^2^ Department of Pharmaceutical Sciences, University of Michigan, Ann Arbor, MI, United States; ^3^ Department of Mechanical Engineering, Bangladesh University of Engineering and Technology, Dhaka, Bangladesh; ^4^ Department of Pediatrics, Division of Pediatric Cardiology, University of Michigan, Ann Arbor, MI, United States; ^5^ Department of Pediatrics, Nationwide Children’s Hospital, Columbus, OH, United States; ^6^ Department of Surgery, University of Michigan, Ann Arbor, MI, United States; ^7^ School of Biomedical Engineering and Imaging Sciences, King’s College London, London, United Kingdom; ^8^ Department of Mechanical Engineering, Michigan State University, East Lansing, MI, United States

**Keywords:** pulmonary arterial hypertension, computational modeling, ventricular-arterial coupling, biomechanics, patient stratification, ventricular mechanics, arterial hemodynamics

## Abstract

Pulmonary arterial hypertension (PAH) is a complex disease involving increased resistance in the pulmonary arteries and subsequent right ventricular (RV) remodeling. Ventricular-arterial interactions are fundamental to PAH pathophysiology but are rarely captured in computational models. It is important to identify metrics that capture and quantify these interactions to inform our understanding of this disease as well as potentially facilitate patient stratification. Towards this end, we developed and calibrated two multi-scale high-resolution closed-loop computational models using open-source software: a high-resolution arterial model implemented using CRIMSON, and a high-resolution ventricular model implemented using FEniCS. Models were constructed with clinical data including non-invasive imaging and invasive hemodynamic measurements from a cohort of pediatric PAH patients. A contribution of this work is the discussion of inconsistencies in anatomical and hemodynamic data routinely acquired in PAH patients. We proposed and implemented strategies to mitigate these inconsistencies, and subsequently use this data to inform and calibrate computational models of the ventricles and large arteries. Computational models based on adjusted clinical data were calibrated until the simulated results for the high-resolution arterial models matched within 10% of adjusted data consisting of pressure and flow, whereas the high-resolution ventricular models were calibrated until simulation results matched adjusted data of volume and pressure waveforms within 10%. A statistical analysis was performed to correlate numerous data-derived and model-derived metrics with clinically assessed disease severity. Several model-derived metrics were strongly correlated with clinically assessed disease severity, suggesting that computational models may aid in assessing PAH severity.

## 1 Introduction

Pulmonary arterial hypertension (PAH), defined by a mean pulmonary arterial pressure greater than 20 mmHg (Simonneau et al., 2019), is a complex disease that causes functional and structural changes in the pulmonary circulation and right ventricle (RV). Ventricular-arterial interactions play an important role in the progression of PAH, where increases in resistance and decreases in compliance of the pulmonary circulation lead to structural remodeling and increased contractility of the RV, in an attempt to maintain normal cardiac outputs (Vonk Noordegraaf et al., 2019). RV contractility can increase four- to five-fold until the ventricle cannot compensate for further increases in pulmonary pressures, leading to “uncoupling” between the RV and pulmonary arteries. Consequently, RV stroke volume and ejection fraction decrease, ultimately leading to decompensated RV failure (Vonk Noordegraaf et al., 2017). Given that ventricular-arterial interactions are a key determinant of the clinical course of PAH (Shimoda and Laurie 2013), there is a pressing need to identify metrics that consider these interactions to accurately describe PAH pathology.

Pediatric PAH is especially difficult to manage as diagnostic metrics are often derived from adult data due to the lack of clinical trials in the pediatric population (Ivy et al., 2010). Using adult population data to guide pediatric PAH treatment can be problematic as younger subjects present significant differences in cardiovascular structure and function (Strait and Lakatta 2012) and show worse survival rates (Douwes et al., 2013). Furthermore, there are population-based differences in children with PAH compared to adults, such as impaired lung development and higher incidence of congenital heart disease (Berger and Bonnet 2010). Clinical trials designed to focus on pediatric PAH patients could help elucidate novel diagnostic metrics for this population.

Computational models based on patient-specific clinical data have widely been used to study mechanics and hemodynamics of cardiovascular diseases (Humphrey and Taylor 2008; Taylor and Figueroa 2009; Nordsletten et al., 2011; Bikia et al., 2020; Miller et al., 2021) including hemodynamics in the cardiopulmonary circulation (Kheyfets et al., 2013). Previous PAH modeling efforts have focused on either the pulmonary arteries (Tang et al., 2012; Kheyfets et al., 2015; Zambrano et al., 2018; Yang et al., 2019) or the RV (Rausch et al., 2011; Avazmohammadi et al., 2019; Shavik et al., 2019). The absence of high-resolution (3D) bi-directional (ventricular-arterial) hemodynamic interactions in these models restricts their ability to capture phenomena such as ventricular-arterial uncoupling. In this work, we aim to overcome this limitation by developing high-resolution (3D) models of both RV and pulmonary artery mechanics, a first step towards a fully 3D bi-directional model of the cardiopulmonary system.

**FIGURE 1:**
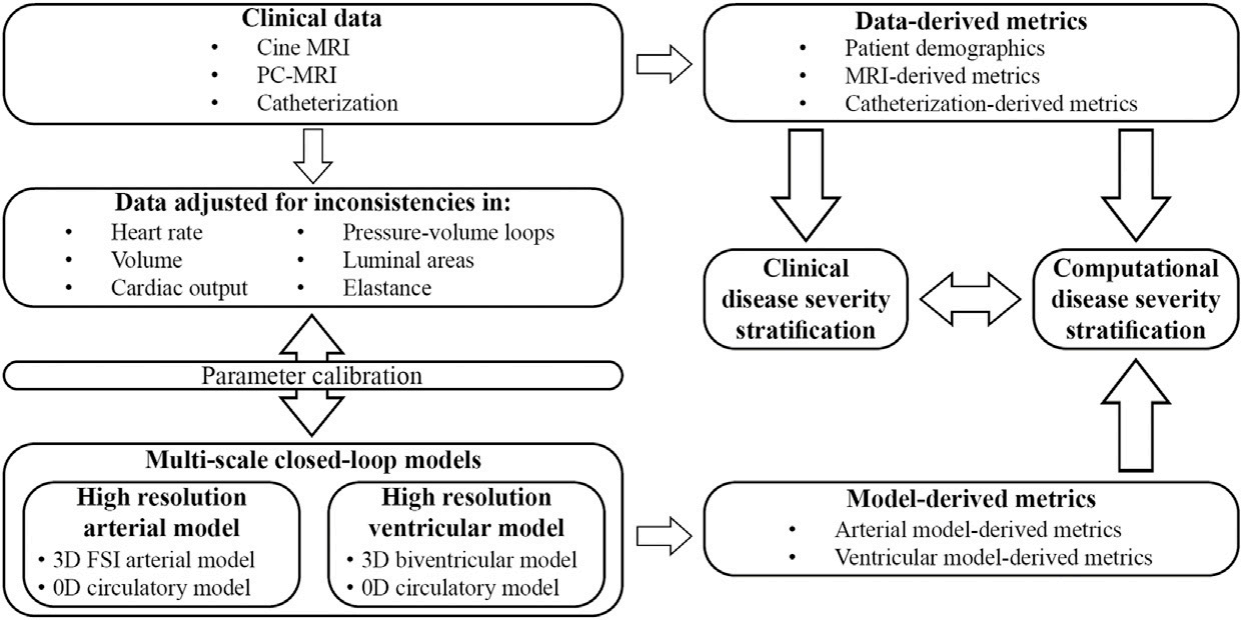
Clinical data was prospectively acquired in pediatric PAH patients and adjusted to mitigate inconsistencies. Parameters of two different closed-loop multiscale models were calibrated and used to study computational metrics of disease severity. Clinically assessed disease severity, data-derived metrics, and model-derived metrics were correlated to stratify patients according to disease severity.

The overall goals of this work are to (Figure 1): (1 develop and calibrate multi-scale closed-loop models of the cardiopulmonary circulation in PAH patients, and (2 use clinical and computational metrics to stratify patients according to disease severity. Two different models will be developed: a “High-Resolution Arterial Model,” whereby image-based 3D fluid-structure interaction (FSI) models of the large vessels are coupled to 0D models of ventricles and distal circulation; and a “High-Resolution Ventricular Model”, whereby image-based 3D models of passive and active ventricular mechanics are coupled to 0D models of arterial and pulmonary circulation. These computational models rely on the quality and consistency of clinical data. Data were acquired using diagnostic tools with varying tolerances, temporal and spatial resolutions, and physiological states (i.e., level of sedation), which led to inconsistencies. In this work, we also propose and implement strategies to mitigate data inconsistencies to inform and calibrate these computational models.

## 2 Materials and methods

Clinical data were acquired prospectively from a cohort of 8 pediatric PAH patients treated at the University of Michigan (UM) C.S. Mott Children’s Hospital (ClinicalTrials.gov ID No. NCT03564522). This study was approved by the UM Institutional Review Board (HUM00117706), and informed consent was obtained from subjects or their parents/guardians. World Health Organization functional class (WHO-FC) was determined for each patient.

### 2.1 Clinical data

Clinical data on anatomy, flow, and pressure, were acquired using MRI and catheterization. Both MRI and catheterization data were acquired with the patient at rest in the supine position. The mean time between MRI acquisition and catheterization was 4.4 days (range 0–29 days).

#### 2.1.1 Magnetic resonance imaging

**FIGURE 2:**
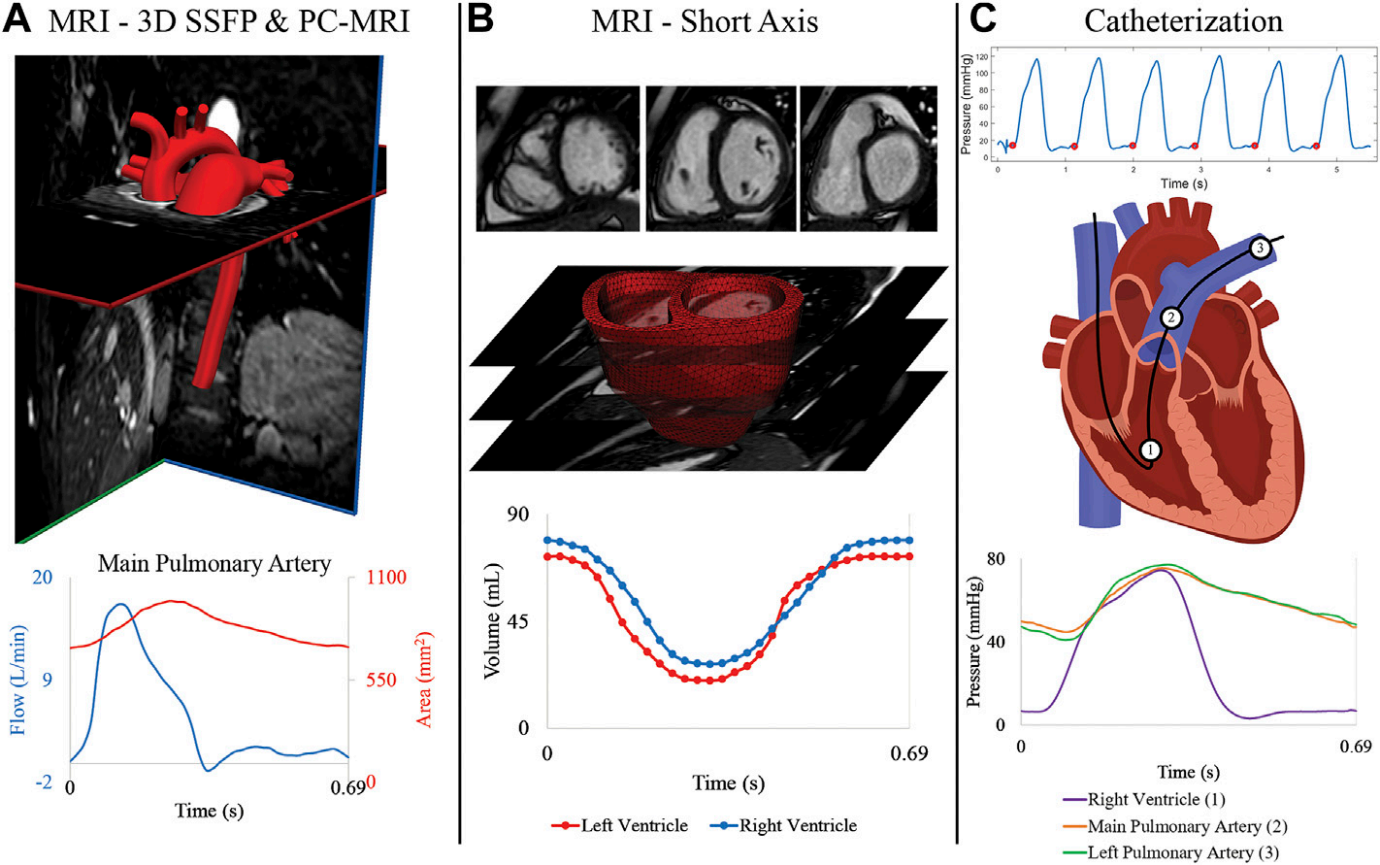
**(A)** MRI 3D SSFP data is used to construct the anatomical arterial models. Flow and area waveforms were reconstructed from PC-MRI data containing 40 phases. **(B)** 30 temporal phases of endocardial and epicardial surfaces were segmented from the short-axis stack of cine MRI data to generate the ventricular volume waveforms. High-resolution (3D) ventricular models were created from these segmented surfaces. **(C)** Pressures at the (1) right ventricle, (2) main pulmonary artery, and (3) right pulmonary artery were acquired with catheterization for all patients.

MRI was performed using a 1.5 Tesla scanner (Achieva or Ingenia; Philips, Best, the Netherlands). Three-dimensional diastolic vascular anatomy was obtained with a 3D steady state free precession (SSFP) sequence (TE: 2.2 ms, TR: 4.3–4.4 ms, flip angle: 90°, field of view: 260–350 mm, slice thickness = 1.4–1.6 mm, image resolution = 0.63–0.78 mm) with cardiac and respiratory gating (Figure 2A). Gated phase-contrast MRI (PC-MRI) (TE: 2.7–3.3 ms, TR: 4.1–5.1 ms, flip angle: 12°, field of view: 250–350 mm, slice thickness = 6 mm, image resolution = 1.4–1.6 mm) was performed to obtain dynamic data at 40 phases of luminal area and blood flow at five anatomical locations: ascending aorta (AAo), descending thoracic aorta (DTA), main pulmonary artery (MPA), left pulmonary artery (LPA), and right pulmonary artery (RPA). Multi-slice (10–13 slices, slice thickness: 6–8 mm), multi-phase (30 phases) cine MR images (TE: 1.2–1.6 ms, TR: 2.5–3.1 ms, flip angle: 60°, field of view: 250–350 mm, image resolution = 1.6–1.8 mm) were acquired by positioning the scans in the short-axis planes perpendicular to the long-axis of the left ventricle (LV), capturing both the LV and RV from the cardiac base to the apex (Figure 2B).

#### 2.1.2 Catheterization

Right heart catheterization was performed in all subjects (Figure 2C) to measure invasive hemodynamics and assess PAH progression. Intracardiac pressures were measured in the right atrium, RV, and pulmonary arteries (MPA, and either LPA or RPA) using a balloon wedge catheter (Arrow, Reading, PA) or thermodilution catheter (Edwards Lifesciences, Irvine, CA). Pulmonary capillary wedge pressure was measured, and femoral arterial access was acquired for blood pressure monitoring and blood gas analysis. In select patients with suspected left heart disease, retrograde left heart catheterization was performed using a pigtail catheter (Merit Medical, South Jordan, UT) and direct pressures were measured in the LV, AAo, and DTA. Pulmonary vascular resistance index (PVRi) was calculated by dividing the pressure gradient (mean pulmonary arterial pressure—mean pulmonary capillary wedge pressure) over cardiac index, which was calculated by the Fick principle or thermodilution.

### 2.2 Patient demographics and clinical metrics

Eight PAH patients (age: 11.8 ± 4.4 years; range 6–19 years) underwent catheterization and MRI examinations. Table 1 summarizes mean and standard deviations of basic patient demographics and several key clinical metrics, derived from MRI and catheterization. Five patients were classified as WHO-FC I and three as WHO-FC II. Four patients were on dual PAH therapy, and four on triple PAH therapy.

**TABLE 1:** Summary of patient demographics and clinical metrics (MRI- and cath-derived).

Patient demographics	Subject #1	Subject #2	Subject #3	Subject #4	Subject #5	Subject #6	Subject #7	Subject #8	Average	Std Dev
Age (years)	11	15	10	5	16	11	19	6	11.6	4.8
Gender (M/F)	F	F	M	F	F	M	F	F	N/A	N/A
BSA (m^2^)	1.23	1.66	0.95	0.74	1.44	1.25	1.61	0.88	1.2	0.3
Height (cm)	154	175	123	106.7	152	152	165	121.9	143.7	23.8
Weight (kg)	33.2	56.3	26.9	18.7	49.8	35.2	56.2	23	37.4	14.9
WHO Functional Class	II	I	II	I	I	II	I	I	N/A	N/A
Number of PAH medications	3	2	3	2	2	3	2	3	2.5	0.5
Years since initial diagnosis	5	5	4	1	10	10	6	2	5.3	3.3
MRI-derived metrics										
Aorta - Flow Rate (L/min)	4.24	4.38	3.74	2.41	5.37	4.79	4.90	3.16	4.1	1.0
MPA - Flow Rate (L/min)	4.80	4.89	3.23	2.39	5.34	3.50	4.40	2.95	3.9	1.1
Averaged Cardiac Output (L/min)	4.52	4.63	3.49	2.40	5.36	4.15	4.65	3.06	4.0	1.0
Cardiac Index (L/min/m^2^)	3.7	2.8	3.7	3.2	3.7	3.3	2.9	3.5	3.3	0.4
% of flow to LPA	45%	37%	51%	40%	47%	51%	41%	49%	0.5	0.1
Pulmonary Regurgitant Factor (%)	1%	1%	N/A	1%	1%	2%	8%	0%	2.0%	2.7%
Heart Rate (bpm)	91	77	79	66	83	74	70	73	76.6	7.7
Cardiac Cycle Length (s)	0.662	0.780	0.756	0.905	0.725	0.815	0.856	0.825	0.79	0.08
RV End-Diastolic Volume (ml)	120	141	84	78	114	123	241	90	123.9	52.1
RV End-Systolic Volume (ml)	60	67	33	39	44	60	145	39	60.9	36.1
RV Stroke Volume (ml)	60	74	51	39	71	63	96	51	63.1	17.5
RV End-Diastolic Volume Index (ml/m^2^)	98	85	88	105	79	98	150	102	100.7	21.7
RV End-Systolic Volume Index (ml/m^2^)	49	40	35	53	31	48	90	44	48.7	18.3
RV Stroke Volume Index (ml/m^2^)	49	45	54	53	49	50	60	58	52.1	5.0
RV Ejection Fraction (%)	50	52	61	49	62	51	40	57	52.8	7.2
RV Mass (g)	30	36	23	16	35	21	72	13	30.7	18.7
RV Mass Index (g/m^2^)	24	22	24	22	24	17	45	14	24.0	9.2
Main Pulmonary Artery Stroke Volume (ml)	56	63	41	37	69	48	65	41	52.5	12.4
LV End-Diastolic Volume (ml)	103	124	82	68	112	139	163	72	107.9	33.5
LV End-Systolic Volume (ml)	41	55	30	30	41	60	72	30	44.9	15.9
LV Stroke Volume (ml)	62	69	51	37	71	79	91	42	62.8	18.5
LV End-Diastolic Volume Index (ml/m^2^)	84	75	86	92	78	111	101	82	88.6	12.3
LV End-Systolic Volume Index (ml/m^2^)	33	33	32	41	28	48	45	34	36.7	6.9
LV Stroke Volume Index (ml/m^2^)	50	42	54	50	49	63	57	48	51.6	6.4
LV Ejection Fraction (%)	60	55	63	55	63	57	56	58	58.4	3.3
LV Mass (g)	55	72	43	31	59	53	92	31	54.5	20.6
LV Mass Index (g/m^2^)	45	43	45	42	41	42	57	35	43.9	6.2
Ascending Aorta Stroke Volume (ml)	53	60	51	39	73	67	78	44	58.1	13.8
Sedation	N	N	N	Y	N	Y	N	N	N/A	N/A
Cath-derived metrics										
Pulmonary arterial mean pressure (mmHg)	59.4	29.2	35.1	82.9	47.1	31.2	58.3	20.4	45.4	20.6
Pulmonary arterial pulse pressure (mmHg)	30.6	18.0	35.4	66.2	36.9	26.6	48.0	24.1	35.7	15.3
Pulmonary arterial systolic pressure (mmHg)	74.7	38.2	52.9	116.0	65.5	44.5	82.3	32.5	63.3	27.6
PVR Index (WU m^2^)	16.2	7.3	5.9	23.2	9.9	4.9	16.0	3.3	10.8	6.9
Rp:Rs	0.8	0.4	0.32	0.77	0.55	0.33	0.8	0.2	0.5	0.2
Pulmonary Capillary Wedge Pressure (mmHg)	10	8	15	14	12	14	12	8	11.5	2.7
Cath Heart Rate (bpm)	69	65	78	66	72	65	66	86	71.0	7.6
Cath Cardiac Cycle Length (s)	0.870	0.918	0.770	0.909	0.830	0.920	0.905	0.695	0.9	0.1
PA Oxygen Saturation (%)	80	72	64	60	73	64	70	73	69.5	6.5

### 2.3 Strategies for mitigation of inconsistencies in clinical data

Despite best efforts made to acquire MRI and catheterization data in close temporal proximity, the studies were on average 4.4 days apart. Furthermore, catheterization and MRI were performed under varying levels of sedation. This, together with the different tolerances and temporal resolutions of MRI and catheterization, leads to inconsistencies in the data that must be addressed in order to use the data for simulations, where conservation laws of mass and momentum balance must be satisfied. Data inconsistencies include: 1) LV and RV volume waveforms segmented from cine MRI short-axis data do not match values obtained with PC-MRI, and 2) misaligned ventricular pressure-volume (PV) data. Additional examples of data inconsistencies are detailed in the Supplementary Material.

#### 2.3.1 Ventricular volume waveforms

##### 2.3.1.1 Ventricular volumes derived from short-axis cine MRI data

LV and RV volume waveforms were first obtained by manually segmenting each of the 30 phases of the cine MRI data using the software package CVI42 (Circle Cardiovascular Imaging, Calgary, Canada). Ventricular segmentation is a time-consuming task susceptible to inter- and intra-observer variability (Miller et al., 2013). It required delineation of LV and RV endocardial surfaces from the apex to the mitral and tricuspid valve, respectively, for each of the 30 phases of the MRI data. In the short-axis cine MRI data, slice thickness (6–8 mm) is much larger than the in-plane image resolution (0.80–0.91 mm). Higher variability in RV segmentation is expected since its shape varies significantly throughout the slices (Petitjean and Dacher 2011; Caudron et al., 2012). Furthermore, delineation of the basal regions of the ventricles can be challenging due to the 10%–20% end-systolic shortening (Rogers et al., 1991; Klein et al., 1998) and the motion of the valves along the long-axis. Average RV and LV stroke volumes of 62.8 ± 18.5 ml and 63.1 ± 17.5 ml, respectively, were obtained.

##### 2.3.1.2 Truncated ventricular volumes

A fixed plane below the tricuspid valve was used to define truncated volume data used in the high-resolution ventricular computational models. This truncation yielded average LV and RV stroke volumes of 43.0 ± 14.7 ml and 40.4 ± 14.9 ml, respectively (see Supplementary Table S1 for values for each patient). These volumes are 31% and 36% smaller than those corresponding to the full ventricle. Then, the end-systolic phase was segmented using MeVisLab (www.mevislab.de) to define STL models of LV and RV. These end-systolic segmentations were adjusted to match the CVI42 ventricular volume data.

The discrepancy between full ventricular and truncated volumes used in the high-resolution computational arterial models is accounted for by different model parameters of the lumped parameter heart model (Section 2.4.2). Conversely, the high-resolution computational ventricular models use the truncated definition of the ventricular volumes. The implications of this inconsistency will be discussed in the limitations section.

##### 2.3.1.3 Ventricular volumes derived from PC-MRI data

Integration of PC-MRI flow waveforms at AAo and MPA provide alternative definitions for LV and RV ventricular volume waveforms, respectively. Average PC-MRI-derived RV and LV stroke volumes of 53.8 ± 11.3 ml and 51.1 ± 10.8 ml, respectively, were obtained. This represents a difference of 19% and 23% relative to the values obtained via segmentation of the short-axis cine-MRI data. This difference could be due to patients holding their breath only for the short-axis cine-MRI sequence, which can increase stroke volume (Guz et al., 1987). In Section 2.4.3, we describe how to combine the PC-MRI and short-axis cine MRI definitions of volume waveforms to improve the comparison between simulated and measured AAo and MPA flow waveforms.

#### 2.3.2 Automated alignment of ventricular pressure and volume data

**FIGURE 3:**
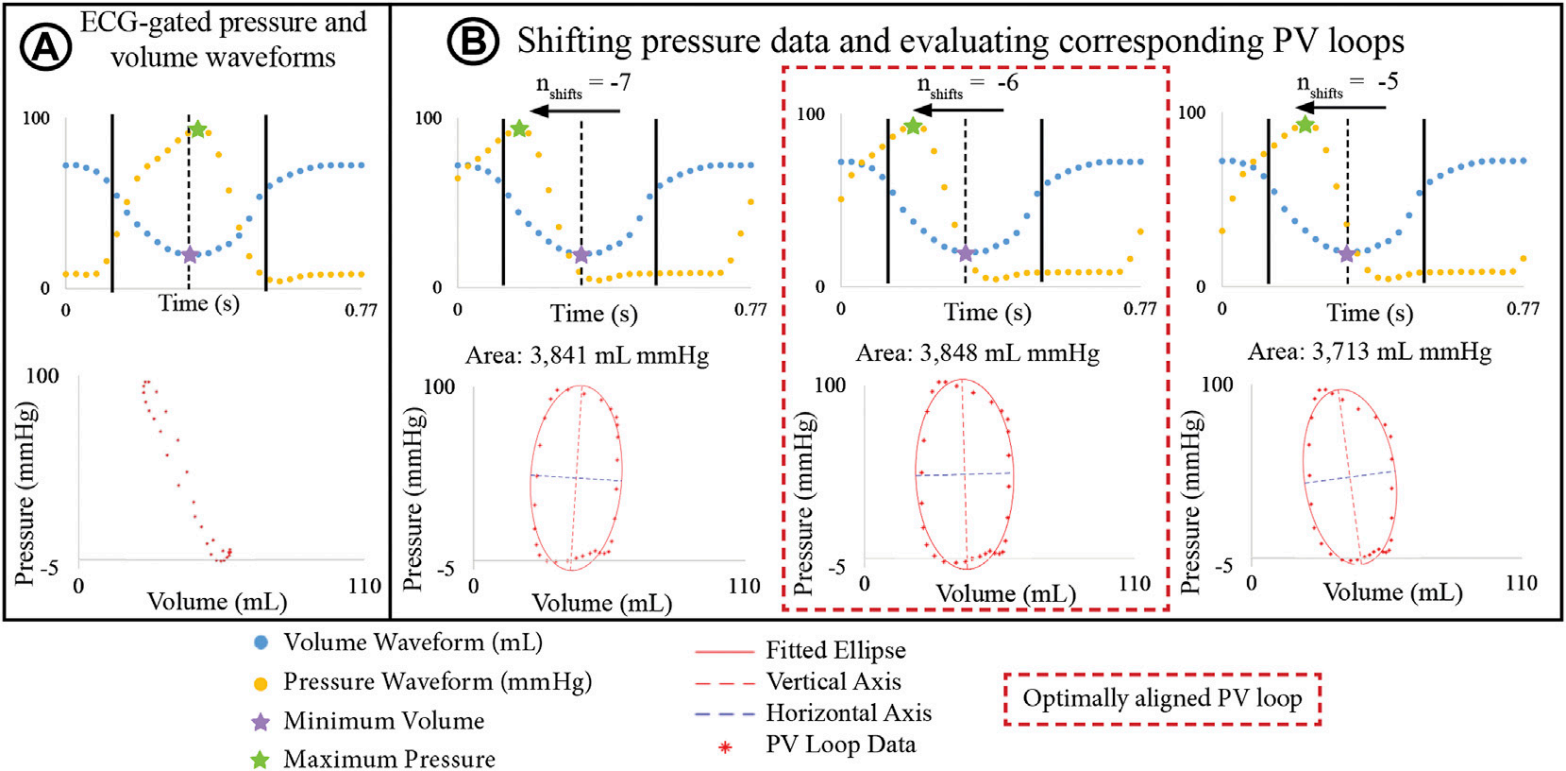
**(A)** PV loop built by ECG-aligning pressure and volume waveforms shows physiologically unrealistic shape. **(B)** Optimization algorithm incrementally shifts pressure waveforms to define a new PV loop and an ellipse is fitted to the PV. The optimally aligned PV loop, showing clearly defined isovolumetric relaxation and contraction phases, is that with the largest area.

Ventricular pressure and volume waveforms were combined to form a PV loop. Given the difference in temporal resolution, pressure waveforms were down sampled to 30 phases to match the resolution of the volume data. Then, pressure and volume waveforms were aligned using the R-peaks of the ECG and a PV loop was plotted. However, in most cases, the shape of the resulting PV loop lacked defined isovolumetric contraction and relaxation phases (Figure 3A), which can even occur when PV loop data is acquired simultaneously with an interventional cardiac MRI. Gusseva et al. (2021; 2022) developed a biophysical heart model to align PV data. In this work, we developed an algorithm to systematically shift the pressure waveforms to determine optimally aligned PV loops.

The basis of this algorithm is as follows: Pressure data was shifted by time increments Δt = T/30 s, where T is the cardiac cycle length, and 30 is the number of phases. Since maximum pressure and minimum volume must remain in close temporal proximity, the number of Δt increments ( ${\text{n}}_{\text{shifts}}$ ) was limited to ±7 (e.g., ${\text{n}}_{\text{shifts}}$ = ± 1, 2, …, 7). Positive and negative signs indicate forward and backwards shifting of the pressure, respectively. For each pair of volume and shifted pressure data, a new PV loop was defined, and an ellipse fitted to the data by least squares minimization (Gal 2020). The optimally aligned PV loop corresponded to the time shift which produced the fitted ellipse with the maximum area (Figure 3B). The optimization algorithm was used to determine the optimally aligned PV loops for both the LV and RV. While the choice of maximum area to identify the optimally aligned PV is arbitrary, it provides a systematic criterion to perform PV loop alignment for every subject.

The aligned PV loops were used to estimate ventricular stroke work, construct the elastance waveforms of the lumped parameter (0D) heart model used in combination with the high-resolution arterial models (Section 2.4.2), and to calibrate the parameters of the high-resolution ventricular models (Section 2.5).

### 2.4 High-resolution arterial model

**FIGURE 4:**
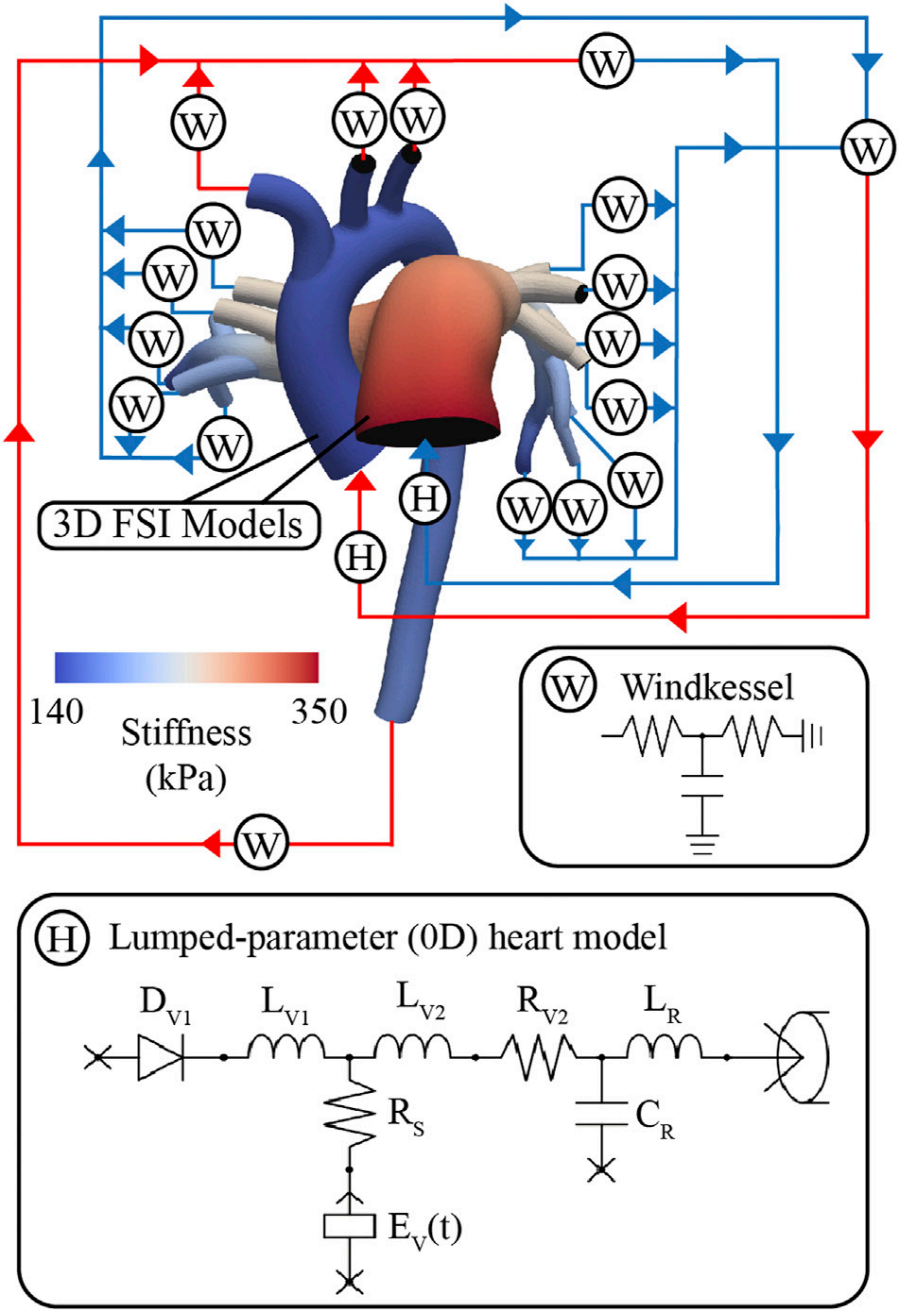
Multi-scale closed-loop model consisting of high-resolution (3D) arterial models of aorta and large pulmonary arteries, coupled to (0D) lumped parameter models of heart (H) and distal circulations (W).

These models consist of a 3D FSI component representing the large systemic and pulmonary arteries, and 0D lumped-parameter models representing the heart (H) (see Figure 4) and the distal systemic and pulmonary circulations via 3-element Windkessel models (W) (Vignon-Clementel et al., 2006).

#### 2.4.1 3D FSI arterial models

Anatomical models of the large systemic and pulmonary arteries were constructed from the diastolic phase of the 3D steady-state free precession MRI data, adjusted to match diastolic areas measured with PC-MRI (Alastruey et al., 2016), see Supplementary Material. Models were built using the open-source cardiovascular simulation software CRIMSON (Arthurs et al., 2021), rendering smooth analytical (NURBS) surfaces enclosing the volume of the vessels of interest. Models were then discretized into linear tetrahedral elements, and field-based adaptivity was used to refine the mesh in regions of high velocity gradients (Sahni et al., 2006). A mesh sensitivity analysis was performed to ensure mesh independence in flow and pressure waveforms, and determine the mesh sizes. The combined finite element mesh size for the aortic and pulmonary models ranged from 1,214,922 to 1,780,093 elements.

The arterial wall was modeled as a linear elastic membrane with spatially varying isotropic stiffness and wall thickness (Figueroa et al., 2006). Luminal area and pressure data were used to derive linearized stiffness, defined as (Hirai et al., 1989; Silva Vieira et al., 2018):

$$E=\frac{1.5\cdot \Delta P\cdot R_{i}^{2}\cdot R_{O}}{\left(R_{O}^{2}-R_{i}^{2}\right)\cdot \Delta R}$$ (1)

where ${\text{R}}_{\text{i}}$ and ${\text{R}}_{\text{O}}\;$ are the diastolic luminal and outer vessel radius, respectively. $\Delta \text{R }(={\text{R}}_{\text{systolic}}-{\text{R}}_{\text{diastolic}}$ ) is the variation in lumen radius, and $\;\Delta \text{P}={\text{P}}_{\text{systolic}}-{\text{P}}_{\text{diastolic}}$ is the pulse pressure. A 15% ratio of wall thickness to vessel radius was used in the large systemic (Roccabianca et al., 2014) and pulmonary (Li et al., 2012) arteries. Linearized stiffness was assessed in the following 5 locations where arterial wall deformation was estimated using PC-MRI: AAo, DTA, MPA, LPA, and RPA. The stiffness values were then linearly interpolated along the vessel centerline. Stiffness in branches was set to match that of the closest large arterial vessel. Stiffness values in each location for each subject are detailed in Supplementary Table S2.

Once the parameters of 3D and 0D compartments of model are defined, multi-scale FSI simulations were performed using the CRIMSON flow solver to solve for the Navier-Stokes equations for an incompressible Newtonian fluid (Figueroa et al., 2006; Xiao et al., 2013; Lau and Figueroa, 2015). All simulations were performed using a time step size of 0.1 ms. Blood was modeled as an incompressible Newtonian fluid with density of *ρ* = 0.00106 g/mm^3^ and viscosity of μ = 0.004 g/mm∙s. Simulations were run until flow and pressure fields achieved cycle-to-cycle periodicity.

#### 2.4.2 Lumped-parameter (0D) heart model

A lumped-parameter (0D) heart model (H) was defined using CRIMSON’S Netlist Editor Boundary Condition Toolbox (Arthurs et al., 2017) (Figure 4). The lumped-parameter heart model used in this work, developed by Kim et al. (2009), captures how changes in either cardiac or arterial properties influence each other. This model was chosen as it was developed by our group, it is implemented in the CRIMSON flow solver, and it has been widely adopted by the 3D hemodynamic modeling field (Sankaran et al., 2012; Marsden 2013; Arthurs et al., 2016; van Bakel et al., 2019). Resistors ( $R_{R}$ ) and capacitors ( $C_{R}$ ) were used to represent the aortic and pulmonary artery roots. Mitral and tricuspid valves were modeled using diodes ( $D_{V1}$ ) and inductors ( $L_{V1}$ ) with set values. Aortic and pulmonary valves were modeled using dynamically controlled resistors ( $R_{V2}$ ) and inductors ( $L_{V2}$ ) (Mynard et al., 2012; Ahmed et al., 2021). These valve models made it possible to reproduce post-systolic flow reversal and non-zero diastolic flow, features both present in our patient cohort.

LV and RV contractility was represented via a time-varying pressure volume chamber representing ventricular elastance ${\text{E}}_{\text{V}}\left(\text{t}\right)$ and a dynamic source resistance ( ${\text{R}}_{\text{S}}$ ) in series. An analytical ‘two-Hill’ function (Mynard et al., 2012) was used to define a smooth ventricular elastance ${\text{E}}_{\text{V}}\left(\text{t}\right)\;$ , whose parameters were adjusted to fit the clinical values of elastance ${\text{E}}_{\text{i}}={\text{P}}_{{\text{V}}_{\text{i}}}/{\text{V}}_{{\text{V}}_{\text{i}}},\text{ i}=1,\;.,30$ defined from the optimally-aligned PV-loops for each patient (Section 2.3.2) (Supplementary Figure S1):

$$E_{V}\left(t\right)=k\left(\frac{g_{1}}{1+g_{1}}\right)\left(\frac{1}{1+g_{2}}\right)+E_{min}$$ (2)

where

$$g_{1}={\left(\frac{t}{\tau_{1}}\right)}^{m_{1}},\;\;g_{2}={\left(\frac{t}{\tau_{2}}\right)}^{m_{2}},\;\;k=\frac{E_{max}-E_{min}}{\max\left[\left(\frac{g_{1}}{1+g_{1}}\right)\left(\frac{1}{1+g_{2}}\right)\right]}$$ (3)

k is a scaling factor, ${\text{m}}_{1}$ and ${\text{τ}}_{1}$ and ${\text{m}}_{2}$ and ${\text{τ}}_{2}$ control the slope and time translation of the ascending and descending portions of the elastance waveform, respectively.

#### 2.4.3 Boundary condition design and calibration

**FIGURE 5:**
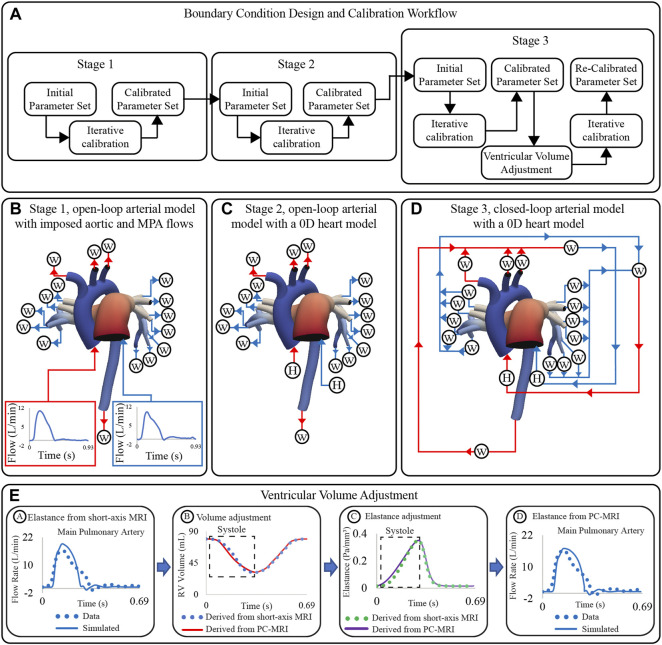
**(A)** Workflow for boundary condition design and calibration of high-resolution arterial models. **(B)** Stage 1: open-loop arterial model with imposed inflow waveforms. **(C)** Stage 2: open-loop arterial model with 0D heart models. **(D)** Stage 3: closed-loop arterial model with a 0D heart models. **(E)** Strategy for ventricular volume adjustment.

Boundary condition design and calibration are achieved via a process which includes three stages of lumped parameter model circuit design, iterative tuning of parameters, and adjustment of truncated ventricular volume and elastance waveforms (Figure 5A). The boundary condition design consists of the following three stages (Arthurs et al., 2017):

Stage 1 open-loop arterial model with imposed aortic and MPA flows (Figure 5B).
Stage 2 open-loop arterial model with a 0D heart model (Figure 5C).
Stage 3 closed-loop arterial model with a 0D heart model (Figure 5D).

Within each stage, parameters were iteratively tuned until simulated results matched clinical data on flow and pressure (Xiao et al., 2014). Calibrated parameters were transferred to the corresponding lumped parameter circuits of the subsequent stage.

Following the three-stage parameter calibration, simulated inflow waveforms did not match the shape of the measured clinical flow waveforms (even though the mean values did). Specifically, simulated inflow waveforms underestimated the length of systole, which led to an overestimated peak systolic flow (Figure 5E, stage A). To fix this discrepancy, PC-MRI data were used to re-define the ventricular volume during systole (Figure 5E, stage B). The adjusted volume waveforms were used to define a new ventricular elastance (Figure 5E, stage C), and simulations with the Stage 3 circuit design were re-run. The new computed inflow waveforms reproduced better the shape of the clinical waveforms (Figure 5E, stage D). A detailed description of the calibration process is given next.

##### 2.4.3.1 Iterative tuning of lumped parameters

Lumped parameter values (Table 2) were estimated using a fixed-point iteration algorithm (Xiao et al., 2014; Alastruey et al., 2016). The iterative algorithm laid out by Xiao et al., (2014) leverages their 1D models to efficiently calibrate outflow boundary condition parameters in 3D patient-specific arterial models. This work was used to define the iterative formulas presented below that aim to match simulated results with clinical hemodynamic data.

**TABLE 2:** Hemodynamic metrics and tuned parameters of the high-resolution arterial model.

Hemodynamic metrics & features	Tuned Parameter(s)
Pulmonary Arterial Mean Pressure	$R_{T}^{Pulmonary}$ $P_{initial}^{Pulmonary}$
Pulmonary Arterial Pulse Pressure	$C_{T}^{Pulmonary}$
Pulmonary Cardiac Output	$R_{T}^{Pulmonary}$ $P_{initial}^{Pulmonary}$
Shape of MPA Flow Waveform	$K_{S}^{RV}$
Systemic Arterial Mean Pressure	$R_{T}^{Systemic}$ $P_{initial}^{Systemic}$
Systemic Arterial Pulse Pressure	$C_{T}^{Systemic}$
Systemic Cardiac Output	$R_{T}^{Systemic}$ $P_{initial}^{Systemic}$
Shape of AAo Flow Waveform	$K_{S}^{LV}$

*Stage 1, open-loop arterial model with imposed aortic and MPA flows*. 3-element Windkessel models were used to represent the resistance and compliance of the distal vascular bed. Windkessel resistances $R_{j}$ and compliances $C_{j}$ for each outlet branch j were iteratively tuned using:

$$R_{j}^{n+1}=R_{j}^{n}\frac{R_{T}^{n+1}}{R_{T}^{n}},\;\;\;\;C_{j}^{n+1}=C_{j}^{n}\frac{C_{T}^{n+1}}{C_{T}^{n}},$$ (4)

where the total arterial resistance *R*_*T*_ and total arterial compliance *C*_*T*_ were iteratively estimated as:

$$R_{T}^{n+1}=R_{T}^{n}+\;\frac{P_{mean}-P_{mean}^{n}}{Q_{mean}^{n}},$$ (5)

$$C_{T}^{n+1}=C_{T}^{n}\frac{P_{pulse}}{P_{pulse}^{n}},$$ (6)

where n is the iteration counter. Simulated ( $P_{mean}^{n}$ , $P_{pulse}^{n}$ ) and measured pressures ( $P_{mean}$ , $P_{pulse}$ ) were compared at the DTA and MPA.

*Stage 2, open-loop arterial model with a 0D heart model*. Initial nodal pressures of the lumped-parameter heart models and Windkessel models for each branch were iteratively tuned:

$$P_{initial}^{n+1}=P_{initial}^{n}*average\left(\frac{\left(Q_{mean}\right)}{\left(Q_{mean}^{n}\right)},\frac{\left(P_{mean}\right)}{\left(P_{mean}^{n}\right)}\right).$$ (7)

Simulated ( $Q_{mean}^{n}$ ) and measured flow rates ( $Q_{mean}$ ) were compared at the AAo and MPA. Coefficients of the dynamic source resistance ( $R_{S}$ ) were tuned to match the decay curve of the inflow waveforms (Mynard et al., 2012).

*Stage 3, closed-loop arterial model with a 0D heart model*. Systemic and pulmonary venous systems, represented via 3-element Windkessel models, were added to connect the arterial outlets to the atria of the lumped-parameter heart models, creating a closed-loop circulation (Figure 5D). Windkessel resistances (Formulas (4) and (5)), Windkessel compliances (Formulas (4) and (6)), and initial nodal pressures of the lumped-parameter models (Formula (7)) were iteratively tuned.

For each patient-specific model, 30–70 lumped parameter values were calibrated until all relative errors between measured and simulated hemodynamics were below 10%. Relative errors were calculated as $\text{|}\left(H_{i}^{data}-H_{i}^{model}\right)/H_{i}^{data}|*100,$ where $H_{i}=\left\{P_{mean},\;P_{systolic},\;P_{diastolic},Q_{mean}\right\}$ for a cardiac cycle once simulated results achieved cycle-to-cycle periodicity. Calibrated lumped parameter model values are detailed in the Supplementary Material (Arterial-Model-Parameter-Values.xlsx) **.**

##### 2.4.3.2 Adjustment of volume and elastance waveforms

The systolic phase of the PC-MRI flow waveform was integrated over time to derive ejected volume. PC-MRI-derived stroke volume was scaled to match short-axis MRI stroke volume. End-diastolic volume was assigned directly from short-axis MRI data, and the scaled PC-MRI-derived volume guided the systolic phase of the ventricular volume waveform. The filling phase of the volume waveform remained unchanged (Figure 5E, stage B). Adjusted ventricular volume waveforms were used to re-derive elastance waveforms (Figure 5E, stage C). Adjusted ‘Two-Hill’ elastance parameter values are found in Supplementary Table S3.

The discrepancies between clinical and simulated MPA and AAo flow waveforms were evaluated using a L2-norm metric $S=\overset{n}{\underset{i=1}{\sum }}{\left(data_{i}-simulation_{i}\right)}^{2}$ . Overall, following the PV-loop alignment and adjustments of the ventricular volume waveforms outlined earlier, reductions in L2-norm metric of 40% ± 18% at the MPA and 10% ± 9% at the AAo were achieved.

### 2.5 High-resolution ventricular model

**FIGURE 6:**
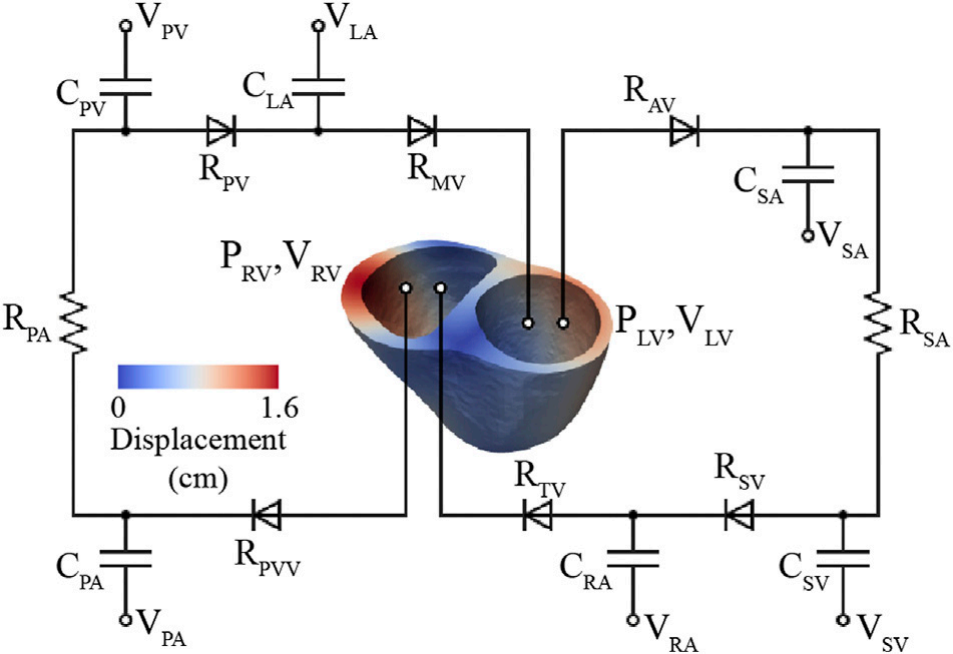
High-resolution (3D) ventricular model coupled to a 0D closed-loop circulatory model, which includes the systemic and pulmonary arteries, venous systems, atria, and valves.

In this model, truncated 3D biventricular geometries (see Section 2.3.1) were coupled to a 0D closed-loop circulatory model representing the distal systemic and pulmonary circulations, atria, and heart valves (Figure 6).

#### 2.5.1 3D biventricular models

Governing equations of the 3D biventricular models based on a quasi-static formulation and assuming that the cardiac tissue behaves as an incompressible material were solved using the open-source software FEniCS (Alnæs et al., 2015), as detailed in Shavik et al. (2019), Shavik et al. (2020), Shavik et al. (2021). An active stress formulation was used to describe the mechanical behavior of myocardial tissue whereby the first Piola Kirchhoff stress tensor P was decomposed into active and passive components $P_{a}$ and $P_{p}$ ( $P=P_{a}+P_{p}$ ), represented with an active contraction model and a passive constitutive model, respectively.

STL models of the truncated ventricles (Section 2.3.1) generated at the end-systolic phase define the unloaded configuration (Hadjicharalambous et al., 2021). This truncation was performed to avoid modeling the basal regions of the heart, including the valves and atria, which are often poorly resolved in the short-axis MRI sequence and are represented with lumped-parameter circuits. The 3D biventricular model was divided into three material regions: LV free wall, septum, and RV free wall. Helix angles of the myocardial fibers were linearly varied along the transmural direction from 60° at the endocardium to −60° at the epicardium (Streeter et al., 1969) using a Laplace-Dirichlet rule-based algorithm (Bayer et al., 2012).

These models were discretized using GMSH (Geuzaine and Remacle 2009) to define tetrahedral grids of 9,159 to 11,726 elements and 2,884 to 3,548 nodes. Mesh refinement studies were performed to ensure that hemodynamic results were independent of mesh size.

##### 2.5.1.1 Active contraction model

The active stress $P_{a}$ was represented using a time-varying elastance model that depends on the length of the myocardial muscle fiber along the local myofiber direction (Guccione et al., 1993; Shavik et al., 2021)

$$P_{a}=T_{ref}\frac{\;Ca_{0}^{2}}{Ca_{0}^{2}+ECa_{50}^{2}}C\left(t\right)\;e_{f}⊗e_{f_{0}}$$ (8)

where $T_{ref}$ is the reference tension, $Ca_{0}$ is the peak intracellular calcium concentration, and $e_{f}$ and $e_{f_{0}}$ are the local vectors that define the muscle fiber direction in current and reference configurations, respectively. The function C(t) is given by

$$C\left(t\right)=\{\begin{array}{c} \frac{1}{2}\left(1-\cos\left(\pi \left(\frac{t}{t_{0}}\right)\right)\right),\;t<t_{t}\\ \frac{1}{2}\left(1-\cos\left(\pi \frac{t_{t}}{t_{0}}\right)\right)\exp\left(\frac{t_{t}-t}{\tau }\right),\;t\geq t_{t} \end{array}$$ (9)

where $t_{0}$ is the time at peak tension, $t_{t}$ is the time at which isovolumic relaxation of the muscle starts, and τ is a time constant associated with the relaxation duration. The length dependent calcium sensitivity $ECa_{50}$ is given by

$$ECa_{50}=\frac{{\left(Ca_{0}\right)}_{max}}{\sqrt{\text{exp}(B\left(l-l_{0}\right)-1)}}$$ (10)

where ${\left(Ca_{0}\right)}_{max}$ is the maximum peak intracellular calcium concentration and $l_{0}$ is the sarcomere length at which no active tension develops.

The choice of active contraction constitutive models in ventricular biomechanics depends on the needs of the study (Wong 1971; Guccione et al., 1993; Niederer et al., 2006; Rice et al., 2008; Chabiniok et al., 2016). In this work, we used an active contraction model based on that from Guccione et al., (1993) which led to the slope of RV ESPVR having a value of 1.32 ± 0.78 mmHg/ml, which is well aligned with values reported in literature (Dell’Italia and Walsh 1988; Brown and Ditchey 1988). Complex active contraction models that consider tropomyosin kinetics (Hunter et al., 1998) or crossbridge cycling (Rice et al., 2008) could have more accurately captured ventricular active mechanics at the expense of more model parameters. However, the modified Guccione et al., (1993) active contraction model is computationally efficient, captures a wide range of myocardial responses, and successfully reproduced clinical data that satisfied the needs of our study.

##### 2.5.1.2 Passive constitutive model

Cardiac tissue is known to be an orthotropic, viscoelastic material (Pinto and Fung 1973; Sommer et al., 2015). Numerous passive constitutive models have assumed a hyper-elastic orthotropic behavior (Holzapfel et al., 2000; Costa et al., 2001; Schmid et al., 2007), and some have included viscoelasticity (Gültekin et al., 2016; Nordsletten et al., 2021; Zhang et al., 2021). Orthotropic viscoelastic models can better represent ventricular biomechanics, at the expense of a larger number of model parameters. For the sake of simpler model parameterization, in this work we have opted for a phenomenological transversely isotropic hyper-elastic model (Guccione et al., 1991; Shavik et al., 2021). In this model, the strain energy function was given by $W=0.5\cdot c\left(e^{Q}-1\right)$ , where Q is a quadratic function of the strain components defined in the material directions:

$$Q=b_{ff}E_{ff}^{2}+b_{xx}\left(E_{ss}^{2}+E_{nn}^{2}+E_{sn}^{2}+E_{ns}^{2}\right)+b_{fx}\left(E_{fn}^{2}+E_{nf}^{2}+E_{fs}^{2}+E_{sf}^{2}\right).$$ (11)

Components of Green-Lagrange strain tensor $E_{ij}$ with (i,j)∈(f,s,n) denote the myocardial fiber (f) , sheet (s) , and sheet normal (n) directions. c is a coefficient scaling passive stiffness. $b_{ff}$ , $b_{xx}$ , and $b_{fx}$ are material constants.

#### 2.5.2 Lumped-parameter (0D) vascular models

The lumped-parameter models of systemic and pulmonary circulation are divided into arterial and venous segments with compliances ( $C_{sa}$ and $C_{sv}$ , $C_{pa}$ and $C_{pv}$ ) and resistances ( $R_{sa}$ and $R_{sv}$ , $R_{pa}$ and $R_{pv}$ ), respectively. Mitral and aortic valves are represented via diodes with resistances ( $R_{mv})$ and ( $R_{av}$ ), respectively. Tricuspid and pulmonary valves are represented via diodes with resistances $\left(R_{tv}\right)$ and ( $R_{pvv}$ ), respectively. Left and right atria were modeled using time-varying elastance functions, respectively (see Figure 6).

#### 2.5.3 Model calibration

##### 2.5.3.1 Parameters of the lumped-parameter vascular models

Parameters were iteratively calibrated until the following simulated and clinical metrics matched within 10%: LV and RV end-diastolic and end-systolic volumes, LV and RV end-systolic pressures, and systemic and pulmonary arterial mean and pulse pressures (Finsberg et al., 2018; Shavik et al., 2019; Shavik et al., 2020), see Table 3. Resting volumes of pulmonary and systemic veins ( $V_{pv,0}$ and $V_{sv,0}$ ) were adjusted to match LV and RV end-diastolic volumes, respectively. However, it should be noted that the impact of tuning $V_{sv,0}$ and $V_{pv,0}$ in most hemodynamic metrics is high (Kass et al., 1986), leading to changes in ventricular end-systolic and end-diastolic pressures and volumes. Systemic and pulmonary arterial resistances ( $R_{sa}$ and $R_{pa}$ ) were adjusted to match systemic and pulmonary arterial mean pressures and flows, and LV and RV end-systolic volumes, respectively. Systemic and pulmonary arterial compliances ( $C_{sa}$ and $C_{pa}$ ) were adjusted to match systemic and pulmonary arterial pulse pressures, respectively. Therefore, LV and RV systolic pressures were also matched by simultaneously calibrating $R_{sa}$ , $R_{pa}$ , $C_{sa}$ and $C_{pa}$ . Parameters of the left and right atrial elastance waveforms were set based on a previous study (Shavik et al., 2019).

**TABLE 3:** Hemodynamic metrics and tuned parameters of the high-resolution ventricular models.

Hemodynamic metrics & features	Tuned Parameter(s)
LV End-Systolic Volume	$T_{ref,LV}$ $V_{pv,0}$ $R_{sa}$
LV End-Systolic Pressure	$R_{sa}$ $T_{ref,LV}$ $C_{sa}$ $V_{pv,0}$
LV End-Diastolic Volume	$V_{pv,0}$
LV End-Diastolic Pressure	$V_{pv,0}$ $C_{LV}$
Systemic Arterial Mean Pressure	$R_{sa}$
Systemic Arterial Pulse Pressure	$C_{sa}$
RV End-Systolic Volume	$T_{ref,RV}$ $V_{sv,0}$ $R_{pa}$
RV End-Systolic Pressure	$R_{pa}$ $T_{ref,RV}$ $C_{pa}$ $V_{sv,0}$
RV End-Diastolic Volume	$V_{sv,0}$
RV End-Diastolic Pressure	$V_{sv,0}$ $C_{RV}$
Pulmonary Arterial Mean Pressure	$R_{pa}$
Pulmonary Arterial Pulse Pressure	$C_{pa}$
Time to Peak Tension	$t_{0}$
Start Time of Relaxation	$t_{t}$
Rate of Relaxation	τ

##### 2.5.3.2 Parameters of the 3D biventricular models

LV free wall and septum were assigned to have the same passive stiffness $C_{LV}$ and contractility $T_{ref,LV}$ values, while RV free wall had distinct values of passive stiffness $C_{RV}$ and contractility $T_{ref,RV}$ . Passive stiffness parameters (*C*_*LV*_ and *C*_*RV*_) were adjusted to match LV and RV end-diastolic pressures, respectively. LV and RV end-systolic volumes were matched by adjusting regional contractility parameters of the active contraction model $T_{ref,LV}$ and $T_{ref,RV}$ , respectively. Parameters of the active contraction model (*t*_*0*_, *t*_*t*_, and τ ) were adjusted to match LV and RV volume and pressure waveforms. The parameter *t*_*0*_ was adjusted to match the time to peak tension, *t*_*t*_ was adjusted to specify the start of the isovolumic relaxation phase and τ was adjusted to match the rate of relaxation of the myofibers.

For each patient-specific model, 16 parameters related to the lumped-parameter vascular models, active contraction models, and passive constitutive models were calibrated and are detailed in Supplementary Table S4.

### 2.6 Metrics for disease severity stratification

Patients were stratified from lowest to highest disease severity, by a team composed of 3 pediatric cardiologists with extensive experience in treating PAH, based on the available clinical metrics (see Table 4). We explored the correlation between the disease severity stratification and clinical metrics and model-derived metrics (from both high-resolution arterial and ventricular models). For each metric, the correlation was assessed using Spearman’s rank correlation coefficient ρ .

**TABLE 4:** Stratification of patients from lowest (value of 1) to highest (value of 8) disease severity.

Clinical disease severity ranking	1	2	3	4	5	6	7	8
Subject Number	Subj. #8	Subj. #2	Subj. #3	Subj. #6	Subj. #5	Subj. #4	Subj. #1	Subj. #7

#### 2.6.1 Clinical metrics

A total of 36 data-derived metrics were evaluated in the disease severity stratification study, including patient demographics (age, height, weight, etc.), indexed MRI-derived metrics (ventricular volume indices, ventricular mass indices, cardiac index etc.), and catheterization-derived metrics (pulmonary arterial pressures, pulmonary vascular resistance, etc.), see Table 1.

#### 2.6.2 Model-derived metrics

A total of 21 model-derived metrics and parameters were included in the disease severity stratification study. The inclusion of the parameters and metrics was determined by the following criteria:

(1) Lumped-parameter model and 3D parameters with a direct counterpart in the clinical data (i.e., total arterial compliance and resistance, linearized arterial stiffness).
(2) All 3D ventricular model parameters (i.e., ventricular contractility and passive stiffness).
(3) Model metrics with well-established clinical meaning such as arterial pulse wave velocity and ventricular-vascular coupling (i.e., RV ESPVR, RV Ea, RV ESPVR/Ea).

Individual lumped parameters for which there were no direct measurements (i.e., proximal and distal resistances, venous circuit parameters, etc.) were excluded.

##### 2.6.2.1 High-resolution arterial models

Stiffness derived at five anatomical locations (AAo, DTA, MPA, LPA, and RPA), peak LV and RV elastance (Section 2.4.2), MPA-LPA, MPA-RPA, and AAo-DTA pulse wave velocities, and distribution of central and peripheral pulmonary vasculature resistance and compliance (Cuomo et al., 2019) were evaluated.

##### 2.6.2.2 High-resolution ventricular models

**FIGURE 7:**
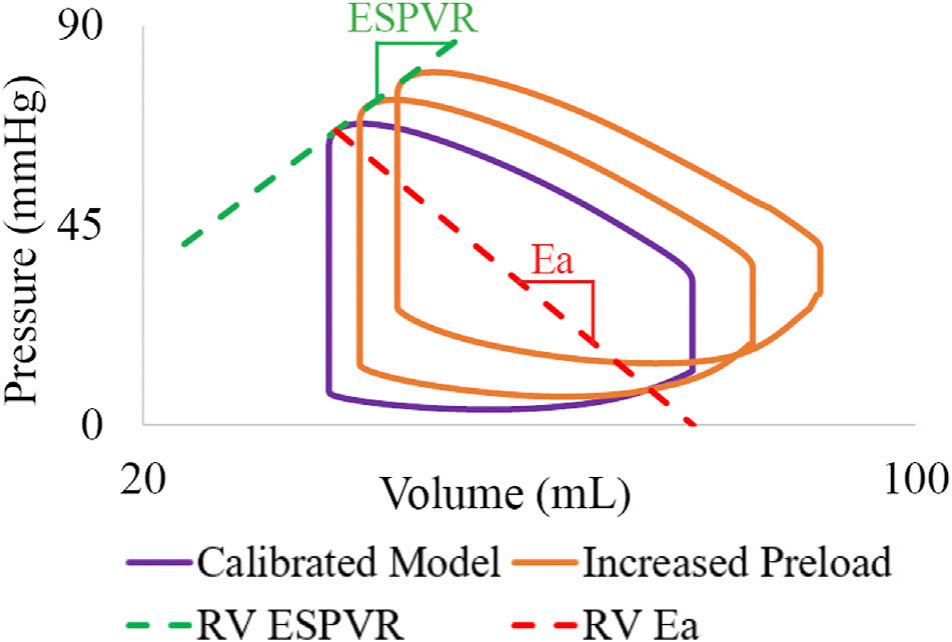
Metrics derived from the high-resolution ventricular models.

RV end-systolic pressure volume relationship (ESPVR), arterial elastance (Ea), and (ESPVR/Ea) ratio were evaluated. For each calibrated subject, RV ESPVR was estimated by varying preload ( $V_{sv,0}$ ) and calculating the slope between peak end-systolic elastance (Figure 7). RV Ea was estimated by dividing end-systolic pressure over stroke volume. Calibrated values of LV and RV contractility ( $T_{ref,LV}$ and $T_{ref,RV}$ ) and passive stiffness ( $C_{LV}$ and $C_{RV}$ ) were also evaluated.

All clinical and model-derived metrics included in the disease severity stratification study are detailed in Supplementary Table S5.

## 3 Results

### 3.1 High-resolution arterial models

**FIGURE 8:**
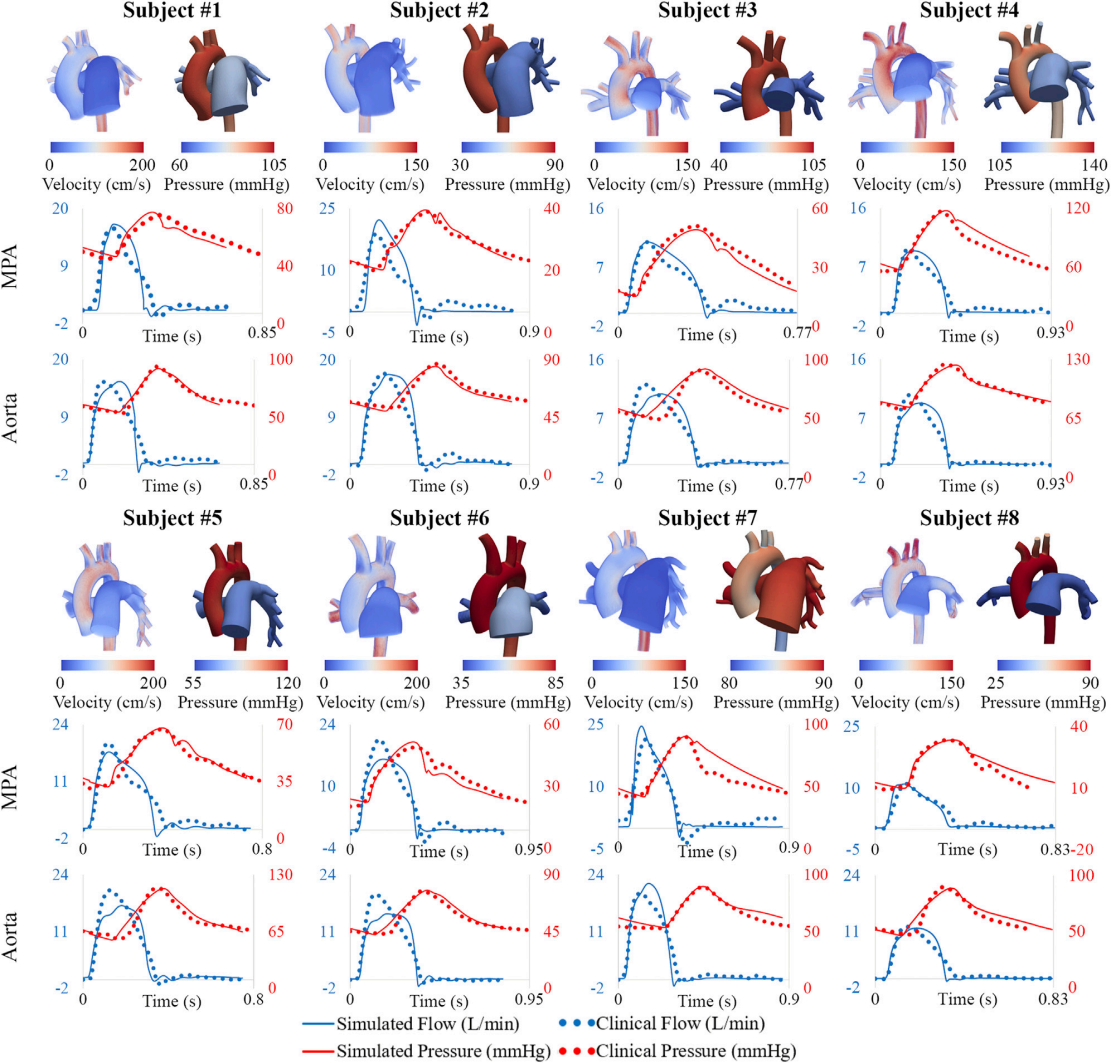
Velocity and pressure maps of the high-resolution arterial models at peak systole. Hemodynamic comparison shows agreement between simulated and clinical data.

Following model parameter calibration (see Table 2), simulation results successfully reproduced patient-specific clinical hemodynamic data within 10% (Figure 8). Pressures were compared between our patient cohort and a cohort of healthy pediatric subjects (Douwes et al., 2013). Pulmonary arterial mean pressures (45.4 ± 19.3 mmHg), and pulmonary arterial pulse pressures (35.7 ± 14.3 mmHg) were higher than values measured in healthy pediatric subjects. Mean systemic arterial pressures (69.9 ± 11.6 mmHg) were similar to those measured in healthy pediatric subjects.

**FIGURE 9:**
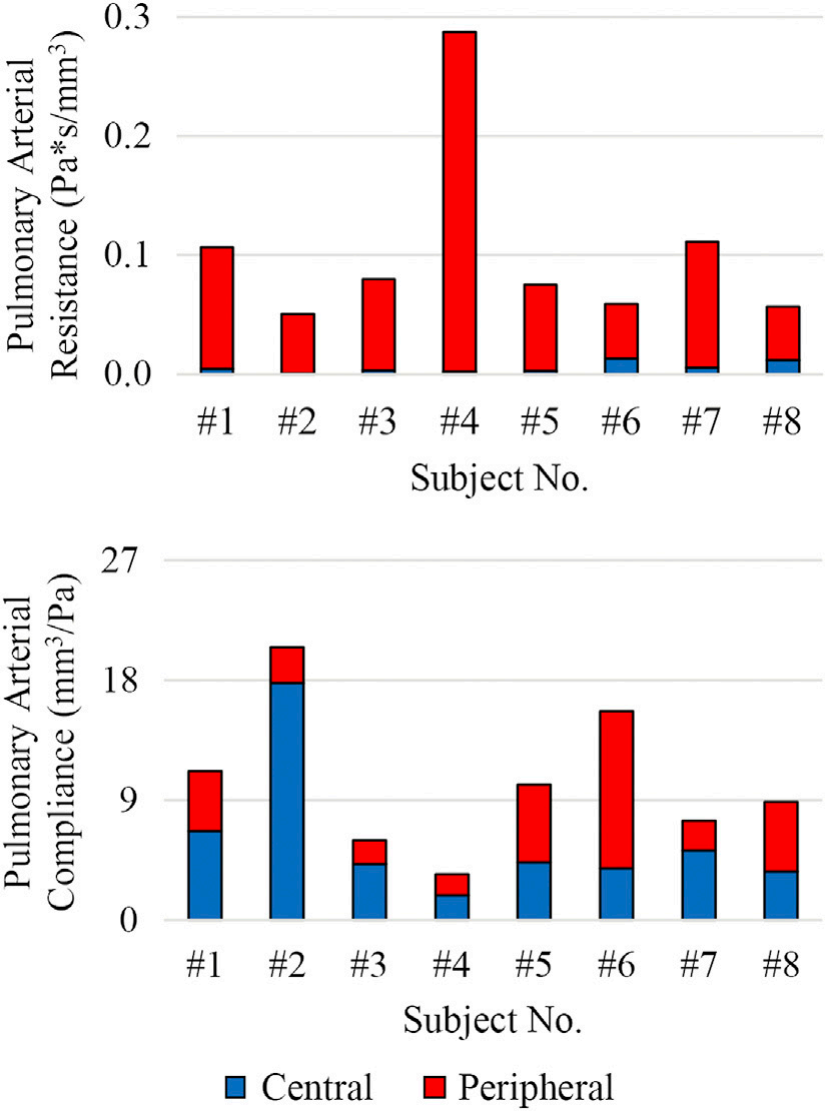
Distribution of total pulmonary arterial resistance and compliance between central (3D model, shown in blue) and peripheral (lumped-parameter models, shown in red) pulmonary vessels.

The distribution of central and peripheral pulmonary vasculature resistance and compliance was obtained. Central (e.g., 3D) pulmonary arteries contributed to 8% ± 8% and 56% ± 19% of the total pulmonary resistance and compliance, respectively (Figure 9).

MPA stiffness (206.6 ± 159.7 kPa) was found to be higher and have a greater variability than AAo stiffness (146.7 ± 19.2 kPa), in line with the severity and disease heterogeneity of these patients. LPA, RPA, and DTA stiffness were estimated to be 163.0 ± 192.3 kPa, 130.4 ± 105.6 kPa, and 227.3 ± 66.4 kPa, respectively.

Pulmonary and systemic arterial pulse wave velocity were estimated to be 3.5 ± 1.5 m/s from the MPA to the LPA, 3.0 ± 0.9 m/s from the MPA to the RPA, and 3.9 ± 1.0 m/s from the AAo to the DTA. Pulmonary arterial stiffness and pulse wave velocity in our patient cohort were higher than in healthy pediatric subjects (Friesen et al., 2019), confirming that our results capture arterial remodeling reflective of PAH.

Subject #4 had the highest pulmonary resistance, lowest pulmonary arterial compliance, highest pulmonary artery stiffness, highest MPA-LPA pulse wave velocity, and near systemic level of pulmonary arterial pressure, indicating that this subject has the most severe form of pulmonary arterial dysfunction. Furthermore, Subject #4 was the only subject with a pulmonary arterial compliance index below the critical threshold value of 0.9 ml/mmHg/m^2^, which has been correlated with a significant reduction in life expectancy (Mahapatra et al., 2006).

### 3.2 High-resolution ventricular models

**FIGURE 10:**
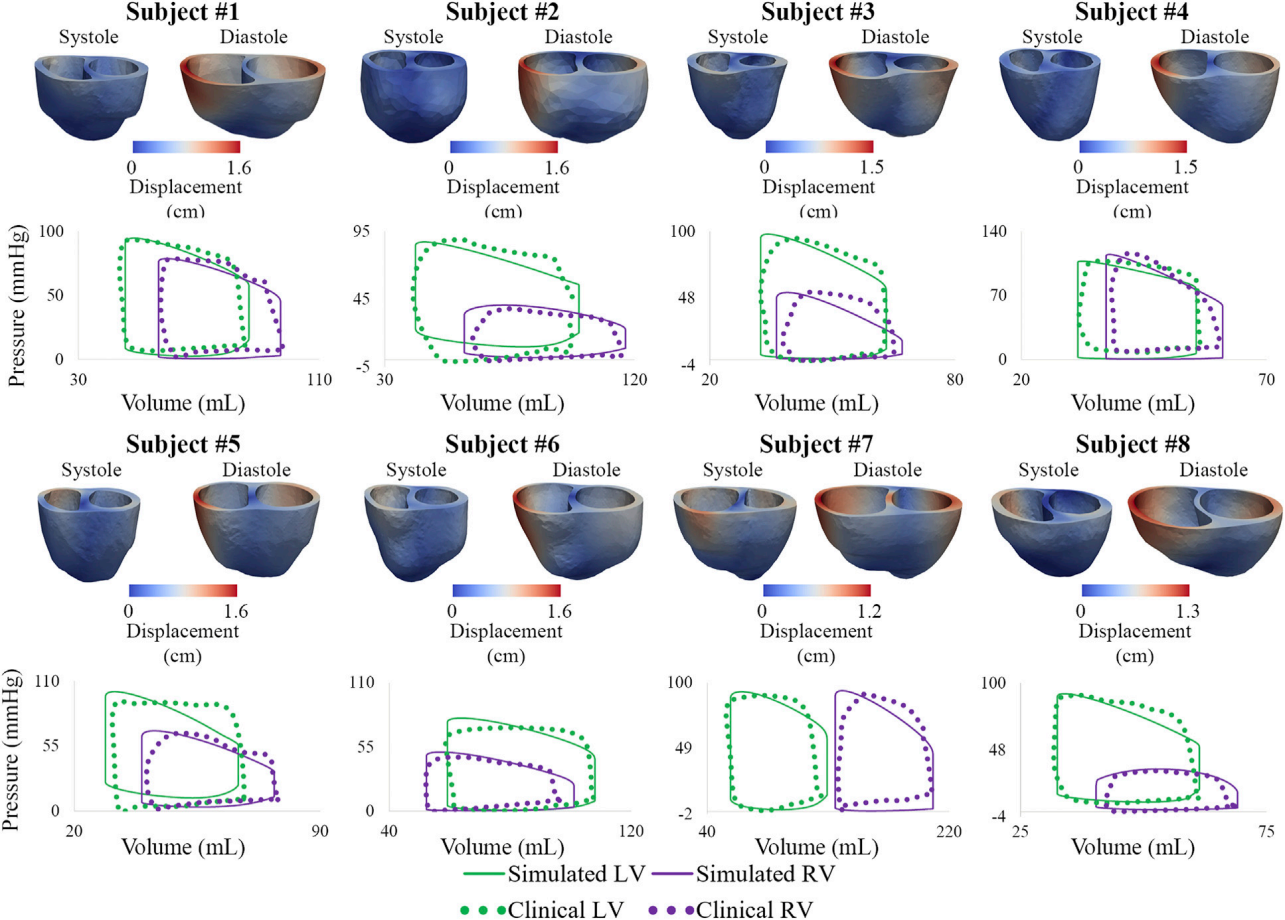
Displacement maps of ventricular models at end-systole and end-diastole. Comparison of LV and RV PV loops shows agreement between simulated and clinical data.

Following model calibration (see Table 3), simulated PV loops and arterial pressures were closely matched to clinical data (Figure 10). On average, RV end-diastolic (99.0 ± 44.6 ml) and end-systolic (58.5 ± 23.4 ml) volumes were larger than their LV counterparts (83.5 ± 23.4 ml and 43.0 ± 14.7 ml, respectively). RV stroke work (0.30 ± 0.17 J) was calculated to be 69% of LV stroke work (0.42 ± 0.14 J). RV ejection fraction (42% ± 5%) was lower than LV ejection fraction (51% ± 5%). Of note, RV ejection fraction in our patients was found to be significantly lower than in healthy pediatric subjects (58% ± 5%) (Friesen et al., 2019), indicating ventricular remodeling.

Calibrated ventricular models were used to derive arterial and RV elastance metrics: ESPVR = 1.32 ± 0.78 mmHg/ml; Ea = 1.84 ± 0.78 mmHg/ml; ESPVR/Ea = 0.75 ± 0.21. RV ESPVR in our cohort was found to be higher than in pediatric patients with repaired tetralogy of Fallot (0.32 ± 0.15 mmHg/ml) (Apitz et al., 2009).

Subject #7 had the largest RV end-diastolic volume, largest RV end-systolic volume (Z-score = 5.4), lowest RV ejection fraction (Z-score = −4.0), and largest RV stroke work (0.71 J), suggesting that this patient had the most severe form of RV dysfunction (Alfakih et al., 2003; Sarikouch et al., 2010). Subject #4, who has the most severe form of pulmonary arterial dysfunction (Section 3.1), has relatively normal RV volumes (Friesen et al., 2019), suggesting that this patient has not undergone significant RV remodeling. These results highlight the importance of simultaneously describing arterial and ventricular hemodynamics and biomechanics.

### 3.3 Metrics for disease severity stratification

The correlation between clinically assessed disease severity (Table 4) and each data- and model-derived metric were ranked using the absolute value of the Spearman’s rank correlation coefficient ρ (Table 5). The analysis was adjusted for multiple comparisons by controlling for a 10% false discovery rate (Benjamini and Hochberg 1995). A metric is significantly correlated if its *p*-value is smaller than its Benjamini-Hochberg critical value (Table 5, iQ/m). After adjusting for multiple comparisons, 13 metrics were found to be significantly correlated, all of which were either catheterization-derived metrics (Table 5; orange) or model-derived metrics (Table 5; green). None of the patient demographics (Table 5; black) or MRI-derived metrics (Table 5; purple) were significantly correlated with disease severity.

**TABLE 5:** Spearman’s rank correlation coefficients (ρ), *p*-values, and Benjamini-Hochberg critical value (iQ/m) of each data-derived and model-derived metric resulting from a comparison to clinical disease severity rankings are shown in two columns. A metric is statistically significant if its *p*-value is lower than its Benjamini-Hochberg critical value (iQ/m).

	Metric	ρ	*p-*value	iQ/m	Metric	ρ	*p-*value	iQ/m
Significantly Correlated Metrics	RPA Stiffness	0.929	<0.001	0.002	RV Systolic Pressure/LV Systolic Pressure	0.857	0.007	0.014
	R_pulmonary/R_systemic	0.929	<0.001	0.003	MPA Diastolic Pressure	0.833	0.010	0.015
	RV Stroke Work/LV Stroke Work	0.905	0.002	0.005	MPA-RPA Pulse Wave Velocity	0.833	0.010	0.018
	RV Contractility (T_Ref,RV_)	0.905	0.002	0.007	LV Contractility (T_Ref,LV_)	−0.810	0.015	0.019
	MPA Systolic Pressure	0.905	0.002	0.009	Total Pulmonary Arterial Resistance	0.810	0.015	0.021
	RV Stroke Work	0.881	0.004	0.010	PVR Index	0.810	0.015	0.023
	MPA Mean Pressure	0.881	0.004	0.012				
Non-Significant Metrics	MPA Stiffness	0.762	0.028	0.025	Age	0.333	0.420	0.063
	MPA Pulse Pressure	0.738	0.037	0.026	Pulmonary Capillary Wedge Pressure	0.286	0.493	0.065
	Pulmonary Arterial Compliance Index	0.738	0.037	0.028	LV End-Systolic Volume Index	0.286	0.493	0.067
	RV Mass Index	0.738	0.037	0.030	Percentage Flow to LPA	−0.286	0.493	0.068
	RV Ejection Fraction	−0.667	0.071	0.032	Catheterization Heart Rate	0.238	0.570	0.070
	MPA-LPA Pulse Wave Velocity	0.643	0.086	0.033	LV Passive Stiffness (C_LV_)	−0.214	0.610	0.072
	MPA Area Index	0.643	0.086	0.035	Height	0.214	0.610	0.074
	RV End-Systolic Volume Index	0.643	0.086	0.037	RV Emax	−0.143	0.736	0.075
	Systemic Arterial Diastolic Pressure	0.619	0.102	0.039	RV Passive Stiffness (C_RV_)	−0.143	0.736	0.077
	Total Pulmonary Arterial Compliance	−0.595	0.120	0.040	RV ESPVR/Ea	−0.119	0.779	0.079
	LPA Stiffness	0.595	0.120	0.042	DTA Stiffness	0.119	0.779	0.081
	RV ESPVR	0.571	0.139	0.044	Weight	0.119	0.779	0.082
	LV Stroke Volume Index	0.524	0.183	0.046	BSA	0.119	0.779	0.084
	LV Mass Index	0.476	0.233	0.047	AAo-DTA Pulse Wave Velocity	0.095	0.823	0.086
	LV End-Diastolic Volume Index	0.476	0.233	0.049	RV SV Index	0.095	0.823	0.088
	MPA Relative Area Change	−0.476	0.233	0.051	Central Pulmonary Arterial Compliance	0.071	0.867	0.089
	Central Pulmonary Arterial Resistance	−0.429	0.289	0.053	MPA Oxygen Saturation	−0.071	0.867	0.091
	RV Ea	0.429	0.289	0.054	AAo Stiffness	−0.048	0.911	0.093
	LV Emax	−0.405	0.320	0.056	MRI Heart Rate	−0.048	0.911	0.095
	Systemic Arterial Mean Pressure	0.381	0.352	0.058	Cardiac Index	0.048	0.911	0.096
	RV End-Diastolic Volume Index	0.381	0.352	0.060	Systemic Arterial Pulse Pressure	0.024	0.955	0.098
	Systemic Arterial Systolic Pressure	0.333	0.420	0.061	LV Ejection Fraction	0.000	1.000	0.010

Patient demographics are shown in black font, MRI-derived metrics are shown in purple font, catheterization-derived metrics are shown in orange font, and model-derived metrics are in green font.

Mean, systolic, and diastolic pulmonary arterial pressures were significantly correlated with clinically assessed disease severity. Pulmonary capillary wedge pressure, which is a measure of the post-capillary pulmonary circulation (i.e., pulmonary venous pressure and LV end-diastolic pressure), was weakly correlated to disease severity (*ρ* = −0.071). Systemic mean, systolic, and diastolic pressures were not significantly correlated to disease severity. There were 5 model-derived metrics (Table 5; green) strongly correlated with disease severity. RV contractility ( $T_{ref,RV}$ ), RPA stiffness, and MPA-RPA pulse wave velocity were all significantly correlated metrics. Interestingly, LV contractility ( $T_{ref,LV}$ ) was the only metric that had a negative, significant correlation with clinically assessed disease severity, suggesting that LV contractility decreases with PAH progression.

## 4 Discussion

Pediatric PAH is a complex disease with a heterogeneous population and multiple compounding factors that contribute to disease progression. It is thus important to identify metrics to stratify patients and to predict disease progression. Computational models enable the study of hemodynamics and biomechanics in the cardiopulmonary and systemic circulations and can be used to describe PAH pathophysiology. In this work, computational models were used to complement clinical data by providing high-resolution description of hemodynamics and biomechanics, including those that are not easy to assess in a clinical setting. To our knowledge, this is one of the first efforts to construct and calibrate two separate high-resolution closed-loop models of pulmonary and systemic arteries and ventricles using data from a pediatric PAH cohort.

Model calibration entailed tuning of numerous model parameters, identifying inconsistencies in clinical data, and developing strategies to mitigate these inconsistencies. Calibrated models could reproduce the following patient-specific data: cardiac output in the MPA and AAo, pressure waveforms at the MPA and DTA, mean flows at the DTA, LPA, and RPA, and LV and RV PV loops. Following calibration, our models were used to derive metrics such as RV ESPVR, arterial elastance (Ea), ESPVR/Ea, ventricular contractility, central pulmonary arterial stiffness and pulse wave velocity, and distribution of pulmonary arterial resistance and compliance between central and peripheral vessels.

Numerous data-derived metrics were correlated with clinical stratification of disease severity. MRI-derived metrics were not significantly correlated with clinical disease severity stratification. The correlation in RV mass index (*ρ* = 0.738) can be attributed to the adaptive response of the RV (myocardial hypertrophy) to a sustained afterload increase, which can also be linked to the strong correlation seen in RV contractility ( $T_{ref,RV}$ ). RV ejection fraction had a correlation value of *ρ* = -0.667, confirming that this commonly used non-invasive metric (Courand et al., 2015; Kjellström et al., 2020) could help in patient stratification. Multiple catheterization-derived (*n* = 8) and model-derived (*n* = 5) metrics had significant correlations with disease severity clinical stratification (Table 5), suggesting the superior specificity of these metrics in capturing PAH disease severity over MRI metrics alone.

RV contractility ( $T_{ref,RV}$ ) had a positive, significant correlation with disease severity, whereas LV contractility ( $T_{ref,LV}$ ) had a negative, significant correlation. This reduction of LV contractility related to PAH progression is commonly attributed to impaired LV diastolic filling (Lazar et al., 1993; Gan et al., 2006) or LV atrophy (Hardziyenka et al., 2011). However, pulmonary capillary wedge pressure (a surrogate for LV diastolic filling pressure) and pulmonary venous resting volume (V_pv,0_) (LV preload parameter) both had weak correlations with disease severity, while LV mass index had a positive, moderate correlation. These suggest that LV diastolic filling and LV mass are not significantly affected by PAH progression. Our results imply, however, that only LV contractility is impaired due to PAH progression which has been confirmed in a computational study comparing PAH patients and control subjects (Finsberg et al., 2019) and in an experimental study where the force-generating capacity of isolated LV cardiomyocites was reduced in PAH patients (Manders et al., 2014).

Our models captured PAH-induced biomechanical adaptations, both in the central (i.e., increase in stiffness and pulse wave velocity) and peripheral (i.e., increase in PVR index) vessels, as well as in the ventricles (i.e., changes in contractility). Increases in pulmonary pressures (i.e., arterial load) were accompanied by increases in RV contractility for the cohort. This suggests that ventricular-arterial coupling was maintained. The weak correlation between model-derived RV ESPVR/Ea and disease severity further supports this observation for the cohort (Vonk Noordegraaf et al., 2017). These findings confirm that computational models of ventricular-arterial interactions can provide additional insight on PAH pathophysiology.

### 4.1 Clinical Applications

The main clinical application areas of our work are two: 1) the potential for replacing or minimizing the number of invasive catheterization procedures in PAH patients, and 2) a more sensitive method for patient stratification.

#### 4.1.1 Potential for minimization of catheter-based assessment in PAH

Computational models required catheterization data and extensive calibration efforts. However, once calibrated using a large cohort of patient data, these computational models could then be validated against new cohorts of patient data without directly inputting measures derived from invasive pressure. This would entail developing correlations between imaging markers such as MPA diameter and relative area change, RV volume, RV ejection fraction, shape of MPA flow waveforms, etc. (Alunni et al., 2010; Lungu et al., 2014; Lungu et al., 2016; Dawes et al., 2017). Furthermore, our calibrated computational models were used to virtually increase ventricular preload to estimate RV ESPVR which obviates the need for an invasive procedure. This model-derived estimation of RV ESPVR can then be used to provide a description of RV contractility and ventricular-arterial coupling (via ESPVR/Ea), which are both known to play key roles in PAH pathophysiology (Vonk Noordegraaf et al., 2019).

#### 4.1.2 Patient stratification in PAH

Despite significant improvements in the understanding of PAH pathological hemodynamics (Kheyfets et al., 2013; Avazmohammadi et al., 2019; Finsberg et al., 2019; Yang et al., 2019), hurdles remain in PAH patient stratification. In this work, computational models were combined with clinical data to stratify PAH patients according to disease severity, confirming well-established data-derived markers (Sanz et al., 2009; Courand et al., 2015; Yang et al., 2018; Simpson et al., 2019) and elucidating model-derived markers that could aid in risk stratification.

PAH requires life-long medical care, and construction and calibration of each computational model typically requires nearly 1 month. This long calibration timeframe could be reduced with advances in data assimilation methods (Troianowski et al., 2011; Ismail et al., 2013; Arthurs et al., 2020). Therefore, patient-specific computational models could be used to provide insight on PAH pathophysiology and stratification and could ultimately help clinicians tailor a long-term management plan.

## 5 Limitations

A small number (*n* = 8) of pediatric PAH patients were included in this study, which affects the strength of the statistical analysis performed to correlate metrics with clinical disease severity stratification. Therefore, due to this small sample size, our analysis was used to broadly describe observed correlations rather than to identify optimal metrics to use in patient stratification. Furthermore, control subjects were not included in our study, which further hinders the ability to identify model parameters for patient stratification. Future extensions of this work will include a greater number of PAH patients as well as control subjects to provide a more robust patient stratification analysis.

A key data inconsistency not addressed in our work was the mismatch in cardiac output between high-resolution arterial and ventricular models. Arterial models were calibrated to match cardiac outputs from PC-MRI data, whereas ventricular models were calibrated to match stroke volumes from truncated ventricular segmentations which yielded lower cardiac outputs. This mismatch stems from truncating the ventricular geometries at the tricuspid valve. Even with this truncation of the ventricular geometries, our ventricular models produced lower RV ejection fraction than those seen in healthy pediatric subjects (Friesen et al., 2019), and outputted metrics that correlated strongly with clinically assessed disease severity.

The number of parameters in our models is much larger than the amount of data used to calibrate our models, which leads to issues of parameter uniqueness and identifiability. However, our choices for the different components of the lumped-parameter circuits are based on well accepted, physiology-motivated, previously developed designs for heart and segments of the circulation (Kim et al., 2009; Lau and Figueroa, 2015; Silva Vieira et al., 2018). The lack of data was offset by (arbitrary) modeling choices such as breakdown of flows proportional to surface areas, ratios of proximal to distal resistances in Windkessel models (Laskey et al., 1990), literature values for certain components of heart models (Mynard et al., 2012; Lau and Figueroa, 2015), etc.

A traditional parameter sensitivity analysis was performed, where the sensitivity of certain computed hemodynamic metrics to 10% changes in model parameters was obtained. The analysis was performed using both the arterial and ventricular models of Subject #6 (Supplementary Tables S6, S7, respectively). The analysis showed that the ventricular models had a larger sensitivity to model parameters than the arterial models. However, this simple analysis does deal address parameter identifiability, as it fails to consider correlation between parameters (Colebank et al., 2021). Therefore, a rigorous sensitivity analysis (outside the scope of this study) that overcomes these limitations is required to assess parameter uniqueness (Matzuka et al., 2015; Perdikaris and Karniadakis, 2016; Caiazzo et al., 2017; Tran et al., 2017; Arthurs et al., 2020; Colebank et al., 2021). This analysis would therefore provide confidence in model-derived metrics and estimated parameters, and also quantify variability in parameters due to uncertainty in clinical data.

Clinically assessed disease severity rankings (Table 4) were used as the gold standard in our disease severity stratification analysis. However, these rankings were based on a combination of hemodynamic metrics and the clinical team’s experience. Even though the clinical team is comprised of pediatric cardiologists with extensive experience in pediatric PAH management, conclusions drawn in our study could be affected by the subjective nature of the disease severity rankings.

High-resolution arterial and ventricular models were not coupled bidirectionally; therefore, parameter calibration in one model does not affect results in the other. The work presented here serves a first step towards the ultimate goal of studying ventricular-vascular coupling in PAH using high-resolution 3D ventricular and arterial models.

## Data Availability

The original contributions presented in the study are included in the article/Supplementary Material, further inquiries can be directed to the corresponding author.
