# Supplementary material for: Data-driven computational models of ventricular-arterial hemodynamics in pediatric pulmonary arterial hypertension
Source: Front Physiol. 2022 Sep 7;13:958734. doi: 10.3389/fphys.2022.958734 (PMC9490558; doi:10.3389/fphys.2022.958734)
Supplement: Supplementary file 1 [file DataSheet1.docx]

Supplementary Material

# Strategies for mitigation of inconsistencies in clinical data

## Heart rate

The average heart rate derived from catheterization (71.0 ± 7.6 bpm) was 7% lower than heart rate derived from PC-MRI (76.6 ± 7.7 bpm). This inconsistency can be attributed to a higher level of sedation used during catheterization than MRI (Janssen et al., 2004). PC-MRI heart rate was deemed more representative of baseline physiologic conditions. Heart rates from the 5 PC-MRI anatomical locations were therefore averaged and used to determine the cardiac cycle lengths for the high-resolution arterial and ventricular simulations.

## Cardiac output

Systemic and pulmonary cardiac outputs measured at the level of the AAo and MPA differed by 5% (4.12 ± 0.98 L/min and 3.94 ± 1.06 L/min, respectively), which are within reported thresholds (Evans et al., 1993). For each patient, representative cardiac outputs were obtained by averaging the systemic and pulmonary measurement. PC-MRI flow waveforms were then scaled accordingly. In Subjects #3, #6, and #7, pulmonary cardiac outputs measured at the MPA were underestimated due to dilation of the MPA causing flow swirling. These underestimated pulmonary cardiac outputs were excluded from the calculations. Averaged cardiac outputs were used to inform the arterial hemodynamic simulations.

## Inferring left ventricular pressure

Since LV pressure data were not measured in most patients, femoral artery and RV pressure waveforms were combined to infer LV pressure. The shape of the LV pressure waveform was assumed to be identical to that of the RV for each patient. To account for the pulse pressure amplification down the aorta, LV systolic pressure was scaled to be 88% of the measured femoral artery systolic pressure (Pauca et al., 2001), whereas the LV diastolic pressure was scaled to match the measured pulmonary capillary wedge pressure. The inferred LV pressure waveforms were then used to generate pressure-volume (PV) loop data used in both arterial and ventricular models.

## Reference diastolic geometry for arterial models

The luminal areas obtained with the diastole-gated 3D SSFP MRI data differed from the 2D PC-MRI diastolic areas at the same location. To ensure consistency between areas when creating the geometric models of aorta and pulmonary arteries (Section 3.4.1), the 3D SSFP areas were scaled to match the 2D PC-MRI diastolic areas (Alastruey et al., 2016).

**Supplementary Table 1.** Truncated ventricular volumes (mL) and ejection fractions (%)

|  | Subject #1 | Subject #2 | Subject #3 | Subject #4 | Subject #5 | Subject #6 | Subject #7 | Subject #8 |
| --- | --- | --- | --- | --- | --- | --- | --- | --- |
| LV End-Diastolic Volume | 86.9 | 97.6 | 63.1 | 56.5 | 69.5 | 107.5 | 125.4 | 61.6 |
| LV End-Systolic Volume | 43.7 | 40.3 | 32.5 | 31.7 | 30.9 | 58.7 | 54.1 | 31.8 |
| LV Stroke Volume | 43.2 | 57.3 | 30.6 | 24.8 | 38.7 | 48.8 | 71.3 | 29.7 |
| LV Ejection Fraction | 50% | 59% | 48% | 44% | 56% | 45% | 57% | 48% |
| RV End-Diastolic Volume | 98.4 | 115.3 | 65.0 | 60.4 | 77.9 | 98.7 | 206.9 | 68.9 |
| RV End-Systolic Volume | 57.4 | 61.9 | 37.8 | 38.4 | 40.7 | 52.2 | 137.4 | 42.5 |
| RV Stroke Volume | 41.0 | 53.4 | 27.3 | 22.1 | 37.3 | 46.5 | 69.5 | 26.4 |
| RV Ejection Fraction | 42% | 46% | 42% | 37% | 48% | 47% | 34% | 38% |

**Supplementary Table 2.** Patient-specific arterial wall stiffness (g/(mm*s²)) calculated from PC-MRI and catheterization data.

|  | Subject #1 | Subject #2 | Subject #3 | Subject #4 | Subject #5 | Subject #6 | Subject #7 | Subject #8 |
| --- | --- | --- | --- | --- | --- | --- | --- | --- |
| Ascending Aorta | 127,574 | 180,633 | 154,465 | 138,415 | 167,320 | 130,646 | 152,662 | 122,186 |
| Descending Aorta | 169,414 | 308,193 | 238,162 | 157,593 | 303,267 | 197,317 | 305,197 | 139,850 |
| MPA | 134,684 | 100,562 | 78,534 | 324,986 | 148,831 | 203,111 | 578,998 | 83,227 |
| LPA | 72,714 | 95,205 | 87,093 | 119,442 | 163,005 | 62,534 | 663,449 | 40,599 |
| RPA | 134,106 | 74,463 | 60,022 | 136,412 | 85,163 | 122,672 | 394,979 | 35,551 |


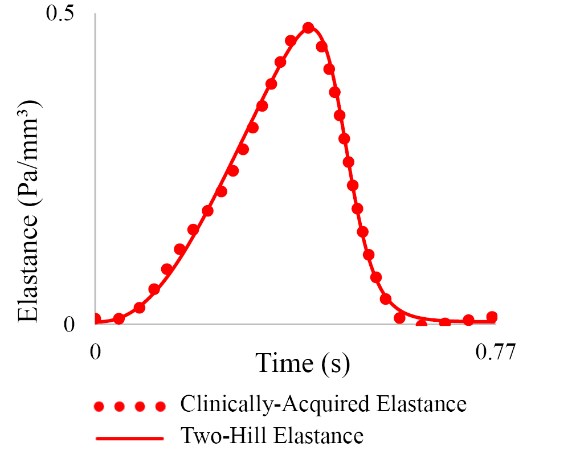


**Supplementary Figure 1.** Patient-specific time-varying elastance function was created using clinically-acquired ventricular pressure and volume (dotted line). An analytical ‘Two-Hill’ elastance function (solid line) was fitted by minimizing least square error to the clinically-acquired elastance waveform. The ‘Two-Hill’ elastance function was used to ensure C^1^ continuity.

**Supplementary Table 3.** Patient-specific elastance waveform parameter values

|  | Subject #1 | Subject #2 | Subject #3 | Subject #4 | Subject #5 | Subject #6 | Subject #7 | Subject #8 |
| --- | --- | --- | --- | --- | --- | --- | --- | --- |
| LV Emax (Pa/(mm^3^)) | 0.236 | 0.334 | 0.451 | 0.637 | 0.336 | 0.177 | 0.168 | 0.370 |
| LV Emin (Pa/(mm^3^)) | 0.015 | 0.015 | 0.010 | 0.030 | 0.019 | 0.009 | 0.009 | 0.022 |
| LV $m_{1}$ | 1.8 | 2.0 | 2.2 | 2.2 | 1.5 | 1.5 | 1.9 | 2.2 |
| LV $\tau_{1}$ | 0.31 | 0.36 | 0.38 | 0.40 | 0.32 | 0.33 | 0.39 | 0.37 |
| LV $m_{2}$ | 18.0 | 24.0 | 16.0 | 24.0 | 24.0 | 24.0 | 20.0 | 22.0 |
| LV $\tau_{2}$ | 0.35 | 0.41 | 0.42 | 0.44 | 0.35 | 0.37 | 0.43 | 0.41 |
| RV Emax (Pa/(mm^3^)) | 0.146 | 0.102 | 0.174 | 0.504 | 0.168 | 0.130 | 0.082 | 0.148 |
| RV Emin (Pa/(mm^3^)) | 0.010 | 0.001 | 0.005 | 0.036 | 0.015 | 0.010 | 0.005 | 0.005 |
| RV $m_{1}$ | 1.6 | 2.0 | 1.7 | 2.3 | 1.6 | 2.0 | 1.7 | 2.1 |
| RV $\tau_{1}$ | 0.28 | 0.38 | 0.38 | 0.44 | 0.35 | 0.35 | 0.37 | 0.39 |
| RV $m_{2}$ | 20.0 | 24.0 | 14.0 | 22.0 | 20.0 | 24.0 | 16.0 | 24.0 |
| RV $\tau_{2}$ | 0.35 | 0.43 | 0.42 | 0.49 | 0.39 | 0.39 | 0.41 | 0.43 |

**Supplementary Table 4.** Calibrated parameter values of patient-specific closed-loop ventricular models

| *Ventricular Model Parameters* | Subject #1 | Subject #2 | Subject #3 | Subject #4 | Subject #5 | Subject #6 | Subject #7 | Subject #8 | Average | Standard Deviation |
| --- | --- | --- | --- | --- | --- | --- | --- | --- | --- | --- |
| C_LV_ (Pa) | 20 | 20 | 20 | 20 | 20 | 30 | 20 | 30 | 23 | 4 |
| C_SEPTUM_ (Pa) | 20 | 20 | 20 | 20 | 20 | 30 | 20 | 30 | 23 | 4 |
| C_RV_ (Pa) | 15 | 15 | 15 | 15 | 15 | 25 | 15 | 15 | 16 | 3 |
| T_0,LV­_ (kPa) | 85 | 125 | 140 | 105 | 110 | 95 | 90 | 110 | 107.5 | 17.1 |
| T_0,SEPTUM­_ (kPa) | 85 | 125 | 140 | 105 | 110 | 95 | 90 | 110 | 107.5 | 17.1 |
| T_0,RV­_ (kPa) | 135 | 60 | 60 | 260 | 110 | 125 | 200 | 34 | 123.0 | 71.5 |
| C_sa_ (Pa) | 0.0060 | 0.0100 | 0.0040 | 0.0047 | 0.0045 | 0.0085 | 0.0115 | 0.0040 | 0.0067 | 0.0028 |
| C_pa_ (Pa) | 0.0070 | 0.0140 | 0.0040 | 0.0025 | 0.0055 | 0.0100 | 0.0085 | 0.0010 | 0.0066 | 0.0040 |
| C_sv_ (Pa) | 0.30 | 0.30 | 0.27 | 0.30 | 0.30 | 0.30 | 0.30 | 0.30 | 0.30 | 0.01 |
| C_pv_ (Pa) | 0.09 | 0.09 | 0.08 | 0.09 | 0.09 | 0.09 | 0.09 | 0.06 | 0.09 | 0.01 |
| R_sa_ (kPa*ms/mL) | 160 | 120 | 240 | 460 | 180 | 130 | 110 | 250 | 206.3 | 107.7 |
| R_pa_ (kPa*ms/mL) | 115 | 10 | 85 | 425 | 60 | 65 | 90 | 13 | 107.9 | 124.6 |
| V_sa,0_ (mL) | 607 | 610 | 700 | 610 | 610 | 610 | 607 | 575 | 616 | 34 |
| V_pa,0_ (mL) | 555 | 500 | 500 | 500 | 100 | 400 | 555 | 525 | 454 | 141 |
| V_sv,0_ (mL) | 3680 | 3450 | 3510 | 3550 | 3300 | 3465 | 3200 | 3380 | 3442 | 140 |
| V_pv,0_ (mL) | 550 | 375 | 405 | 525 | 100 | 470 | 325 | 550 | 413 | 142 |
| Left Ventricular Passive Stiffness (C_LV_), Septum Passive Stiffness (C_SEPTUM_), Right Ventricular Passive Stiffness (C_RV_), Left Ventricular Maximum Tension (T_0,LV_), Septum Maximum Tension (T0,Septum), Right Ventricular Maximum Tension (T_0,RV_), Systemic Arterial Compliance (C_sa_), Pulmonary Arterial Compliance (C_pa_), Systemic Venous Compliance (Csv), Pulmonary Venous Compliance (C_pv_), Systemic Arterial Resistance (R_sa_), Pulmonary Arterial Resistance (R_pa_), Systemic Arterial Resting Volume (V_sa,0_), Pulmonary Arterial Resting Volume (V_pa,0_), Systemic Venous Resting Volume (V_sv,0_), Pulmonary Venous Resting Volume (V_pv,0_) | | | | | | | | | | |

**Supplementary Table 5.** Aggregate of patient demographics, data-derived metrics (MRI- and cath-derived), and model-derived metrics used in the disease severity stratification analysis.

| **Patient Demographics** | Subject #1 | Subject #2 | Subject #3 | Subject #4 | Subject #5 | Subject #6 | Subject #7 | Subject #8 | Average | Std Dev |
| --- | --- | --- | --- | --- | --- | --- | --- | --- | --- | --- |
| Age (years) | 11 | 15 | 10 | 5 | 16 | 11 | 19 | 6 | 11.6 | 4.8 |
| BSA (m²) | 1.23 | 1.66 | 0.95 | 0.74 | 1.44 | 1.25 | 1.61 | 0.88 | 1.2 | 0.3 |
| Height (cm) | 154 | 175 | 123 | 106.7 | 152 | 152 | 165 | 121.9 | 143.7 | 23.8 |
| Weight (kg) | 33.2 | 56.3 | 26.9 | 18.7 | 49.8 | 35.2 | 56.2 | 23 | 37.4 | 14.9 |
|  |  |  |  |  |  |  |  |  |  |  |
| **MRI-derived metrics** | Subject #1 | Subject #2 | Subject #3 | Subject #4 | Subject #5 | Subject #6 | Subject #7 | Subject #8 | Average | Std Dev |
| Cardiac Index (L/min/m²) | 3.7 | 2.8 | 3.7 | 3.2 | 3.7 | 3.3 | 2.9 | 3.5 | 3.3 | 0.4 |
| % of flow to LPA | 45% | 37% | 51% | 40% | 47% | 51% | 41% | 49% | 0.5 | 0.1 |
| Heart Rate (bpm) | 91 | 77 | 79 | 66 | 83 | 74 | 70 | 73 | 76.6 | 7.7 |
| RV End-Diastolic Volume Index (mL/m²) | 98 | 85 | 88 | 105 | 79 | 98 | 150 | 102 | 100.7 | 21.7 |
| RV End-Systolic Volume Index (mL/m²) | 49 | 40 | 35 | 53 | 31 | 48 | 90 | 44 | 48.7 | 18.3 |
| RV Stroke Volume Index (mL/m²) | 49 | 45 | 54 | 53 | 49 | 50 | 60 | 58 | 52.1 | 5.0 |
| RV Ejection Fraction (%) | 50 | 52 | 61 | 49 | 62 | 51 | 40 | 57 | 52.8 | 7.2 |
| RV Mass Index (g/m²) | 24 | 22 | 24 | 22 | 24 | 17 | 45 | 14 | 24.0 | 9.2 |
| LV End-Diastolic Volume Index (mL/m²) | 84 | 75 | 86 | 92 | 78 | 111 | 101 | 82 | 88.6 | 12.3 |
| LV End-Systolic Volume Index (mL/m²) | 33 | 33 | 32 | 41 | 28 | 48 | 45 | 34 | 36.7 | 6.9 |
| LV Stroke Volume Index (mL/m²) | 50 | 42 | 54 | 50 | 49 | 63 | 57 | 48 | 51.6 | 6.4 |
| LV Ejection Fraction (%) | 60 | 55 | 63 | 55 | 63 | 57 | 56 | 58 | 58.4 | 3.3 |
| LV Mass Index (g/m²) | 45 | 43 | 45 | 42 | 41 | 42 | 57 | 35 | 43.9 | 6.2 |
| MPA Diastolic Area (mm^2^) | 586.9 | 477.2 | 308.4 | 493.2 | 281.8 | 529.2 | 879.1 | 382.4 | 492.3 | 176.9 |
| MPA Relative Area Change | 35% | 29% | 75% | 31% | 38% | 20% | 12% | 43% | 35% | 18% |
|  |  |  |  |  |  |  |  |  |  |  |
| **Cath-derived metrics** | Subject #1 | Subject #2 | Subject #3 | Subject #4 | Subject #5 | Subject #6 | Subject #7 | Subject #8 | Average | Std Dev |
| Pulmonary arterial mean pressure (mmHg) | 59.4 | 29.2 | 35.1 | 82.9 | 47.1 | 31.2 | 58.3 | 20.4 | 45.4 | 20.6 |
| Pulmonary arterial systolic pressure (mmHg) | 74.7 | 38.2 | 52.9 | 116.0 | 65.5 | 44.5 | 82.3 | 32.5 | 63.3 | 27.6 |
| Pulmonary arterial diastolic pressure (mmHg) | 44.1 | 20.2 | 17.4 | 49.8 | 28.6 | 17.9 | 34.4 | 8.3 | 27.6 | 13.4 |
| Pulmonary arterial pulse pressure (mmHg) | 30.6 | 18.0 | 35.4 | 66.2 | 36.9 | 26.6 | 48.0 | 24.1 | 35.7 | 15.3 |
| Systemic arterial mean pressure (mmHg) | 68.1 | 65.1 | 66.2 | 97.2 | 77.6 | 56.9 | 65.0 | 63.5 | 69.9 | 11.6 |
| Systemic arterial systolic pressure (mmHg) | 94.0 | 87.0 | 91.0 | 124.9 | 114.3 | 71.1 | 89.1 | 89.8 | 95.1 | 15.7 |
| Systemic arterial diastolic pressure (mmHg) | 53.6 | 52.3 | 49.6 | 75.0 | 56.6 | 46.6 | 52.9 | 47.2 | 54.2 | 8.4 |
| Systemic arterial pulse pressure (mmHg) | 40.4 | 34.7 | 41.4 | 49.9 | 57.6 | 24.5 | 36.2 | 42.5 | 40.9 | 9.3 |
| PVR Index (WU m²) | 16.2 | 7.3 | 5.9 | 23.2 | 9.9 | 4.9 | 16.0 | 3.3 | 10.8 | 6.9 |
| Rp:Rs | 0.8 | 0.4 | 0.32 | 0.77 | 0.55 | 0.33 | 0.8 | 0.2 | 0.5 | 0.2 |
| Pulmonary Capillary Wedge Pressure (mmHg) | 10 | 8 | 15 | 14 | 12 | 14 | 12 | 8 | 11.5 | 2.7 |
| Pulmonary Arterial Oxygen Saturation (%) | 80% | 72% | 64% | 60% | 73% | 64% | 70% | 73% | 70% | 6% |
| Heart Rate (bpm) | 69 | 65 | 78 | 66 | 72 | 65 | 66 | 86 | 71.0 | 7.6 |
| RV Systolic Pressure/LV Systolic Pressure (%) | 79% | 44% | 58% | 93% | 57% | 63% | 92% | 36% | 65% | 20% |
| RV Stroke Work (mmHg mL) | 2227 | 2002 | 2015 | 3277 | 1727 | 2561 | 4758 | 1166 | 2467 | 979 |
| RV Stroke Work/LV Stroke Work (%) | 88% | 51% | 48% | 98% | 72% | 61% | 95% | 42% | 69% | 21% |
| Pulmonary Arterial Compliance Index (mL/mmHg/m^2^) | 0.63 | 0.40 | 0.66 | 1.26 | 0.75 | 0.53 | 0.80 | 0.42 | 0.68 | 0.26 |
|  |  |  |  |  |  |  |  |  |  |  |
|  |  |  |  |  |  |  |  |  |  |  |
| **Arterial model-derived metrics** | Subject #1 | Subject #2 | Subject #3 | Subject #4 | Subject #5 | Subject #6 | Subject #7 | Subject #8 | Average | Std Dev |
| LV Max Elastance (Pa/(mm^3^)) | 0.236 | 0.334 | 0.451 | 0.637 | 0.336 | 0.177 | 0.168 | 0.370 | 0.339 | 0.145 |
| RV Max Elastance (Pa/(mm^3^)) | 0.146 | 0.102 | 0.174 | 0.504 | 0.168 | 0.130 | 0.082 | 0.148 | 0.182 | 0.125 |
| AAo Stiffness (Pa) | 127574 | 180633 | 154465 | 138415 | 167320 | 130646 | 152662 | 122186 | 146738 | 19254 |
| DTA Stiffness (Pa) | 169414 | 308193 | 238162 | 157593 | 303267 | 197317 | 305197 | 139850 | 227374 | 66386 |
| MPA Stiffness (Pa) | 134684 | 100562 | 78534 | 324986 | 148831 | 203111 | 578998 | 83227 | 206617 | 159720 |
| LPA Stiffness (Pa) | 72714 | 95205 | 87093 | 119442 | 163005 | 62534 | 663449 | 40599 | 163005 | 192331 |
| RPA Stiffness (Pa) | 134106 | 74463 | 60022 | 136412 | 85163 | 122672 | 394979 | 35551 | 130421 | 105615 |
| AAo-DTA Pulse Wave Velocity (m/s) | 2.79 | 5.36 | 3.61 | 4.39 | 5.10 | 2.98 | 4.14 | 3.00 | 3.92 | 0.92 |
| MPA-LPA Pulse Wave Velocity (m/s) | 3.51 | 3.14 | 2.19 | 6.30 | 2.93 | 1.88 | 5.58 | 2.31 | 3.48 | 1.52 |
| MPA-RPA Pulse Wave Velocity (m/s) | 4.05 | 2.64 | 2.06 | 3.82 | 2.64 | 1.86 | 4.57 | 2.04 | 2.96 | 0.97 |
| Total Pulmonary Arterial Resistance (Pa*s/mm^3^) | 0.11 | 0.05 | 0.07 | 0.28 | 0.08 | 0.06 | 0.11 | 0.05 | 0.10 | 0.07 |
| Central Pulmonary Arterial Resistance (Pa*s/mm^3^) | 0.0044 | 0.0006 | 0.0035 | 0.0020 | 0.0028 | 0.0132 | 0.0057 | 0.0069 | 0.0049 | 0.0036 |
| Total Pulmonary Arterial Compliance (mm^3^/Pa) | 11.16 | 20.49 | 5.98 | 3.45 | 10.17 | 17.41 | 7.46 | 21.90 | 12.25 | 6.45 |
| Central Pulmonary Arterial Compliance (mm^3^/Pa) | 6.66 | 17.81 | 4.19 | 1.88 | 4.34 | 5.62 | 5.25 | 16.67 | 7.80 | 5.61 |
|  |  |  |  |  |  |  |  |  |  |  |
| **Ventricular model-derived metrics** | Subject #1 | Subject #2 | Subject #3 | Subject #4 | Subject #5 | Subject #6 | Subject #7 | Subject #8 | Average | Std Dev |
| LV Passive Stiffness (Pa) | 20 | 20 | 20 | 20 | 20 | 30 | 20 | 30 | 23 | 4 |
| RV Passive Stiffness (Pa) | 15 | 15 | 15 | 15 | 15 | 25 | 15 | 15 | 16 | 3 |
| LV Active Contractility (T_ref,LV_) (Pa) | 85000 | 125000 | 140000 | 105000 | 110000 | 95000 | 90000 | 110000 | 107500 | 17139 |
| RV Active Contractility (T_ref,RV_) (Pa) | 135000 | 60000 | 60000 | 260000 | 110000 | 125000 | 200000 | 34000 | 123000 | 71479 |
| RV ESPVR (mmHg/mL) | 1.32 | 0.64 | 1.22 | 3.19 | 1.63 | 1.22 | 0.70 | 0.63 | 1.32 | 0.78 |
| RV Ea (mmHg/mL) | 1.96 | 0.73 | 1.69 | 4.86 | 1.80 | 1.03 | 1.32 | 1.33 | 1.84 | 1.20 |
| RV ESPVR/Ea | 0.67 | 0.89 | 0.72 | 0.66 | 0.91 | 1.18 | 0.54 | 0.47 | 0.75 | 0.21 |

**Supplementary Table 6.** Normalized sensitivity values of measured hemodynamic metrics to parameter changes of 10% in the high-resolution arterial model of Subject #6. The normalized sensitivity value represents the ratio of change in error relative to a change in parameter value. For example, a sensitivity value of 1 reflects a 10% change in the metric in response to a 10% change in a given model parameter. Total peripheral resistances and compliances were calculated by combining the effect of Windkessel models, as detailed in (Cuomo et al., 2019).

|  |  | **Computed Hemodynamic Metrics** | | | | | | | | | |
| --- | --- | --- | --- | --- | --- | --- | --- | --- | --- | --- | --- |
|  |  | Pulmonary Arterial Flow Rate | Pulmonary Arterial Systolic Pressure | Pulmonary Arterial Diastolic Pressure | Pulmonary Arterial Pulse Pressure | Pulmonary Arterial Mean Pressure | Aortic Flow Rate | Aortic Systolic Pressure | Aortic Diastolic Pressure | Aortic Pulse Pressure | Aortic Mean Pressure |
| **Parameters** | Total Pulmonary Arterial Resistance | -0.141 | 0.275 | 0.355 | 0.216 | 0.125 | -0.120 | -0.026 | -0.048 | 0.000 | -0.052 |
|  | Total Pulmonary Venous Resistance | 0.006 | 0.120 | 0.329 | -0.037 | 0.228 | -0.087 | -0.065 | -0.162 | 0.050 | -0.099 |
|  | Total Pulmonary Arterial Compliance | 0.077 | 0.003 | 0.213 | -0.155 | 0.062 | -0.155 | 0.017 | 0.111 | -0.096 | 0.012 |
|  | Total Pulmonary Venous Compliance | 0.103 | 0.066 | 0.050 | 0.079 | 0.061 | 0.013 | 0.029 | 0.093 | -0.046 | 0.051 |
|  | RV Source Resistance Coefficient (Ks) | 0.027 | -0.135 | -0.116 | -0.149 | -0.103 | -0.141 | -0.039 | 0.007 | -0.095 | -0.035 |
|  | Pulmonary Vasculature Initial Pressures | 0.386 | 0.429 | 0.485 | 0.386 | 0.454 | 0.349 | 0.293 | 0.328 | 0.251 | 0.330 |
|  | Total Systemic Arterial Resistance | -0.191 | -0.102 | 0.028 | -0.200 | -0.060 | -0.380 | 0.359 | 0.745 | -0.102 | 0.469 |
|  | Total Systemic Venous Resistance | 0.035 | -0.036 | 0.017 | -0.076 | -0.002 | -0.008 | 0.064 | 0.114 | 0.004 | 0.100 |
|  | Total Systemic Arterial Compliance | 0.104 | 0.028 | 0.022 | 0.033 | 0.038 | -0.131 | -0.215 | 0.192 | -0.699 | -0.048 |
|  | Total Systemic Venous Compliance | 0.141 | 0.011 | -0.029 | 0.041 | 0.025 | -0.110 | 0.005 | 0.092 | -0.098 | 0.010 |
|  | LV Source Resistance Coefficient (Ks) | -0.178 | -0.075 | 0.105 | -0.210 | -0.012 | -0.316 | -0.229 | -0.157 | -0.314 | -0.225 |
|  | Systemic Vasculature Initial Pressures | 0.365 | 0.378 | 0.388 | 0.371 | 0.382 | 0.329 | 0.211 | 0.242 | 0.175 | 0.189 |

**Supplementary Table 7.** Normalized sensitivity values of measured hemodynamic metrics to parameter changes of 10% in the high-resolution ventricular model of Subject #6. The normalized sensitivity value represents the ratio of change in error relative to a change in parameter value. For example, a sensitivity value of 1 reflects a 10% change in the metric in response to a 10% change in a given model parameter.

|  |  | **Computed Hemodynamic Metrics** | | | | | | | | | |
| --- | --- | --- | --- | --- | --- | --- | --- | --- | --- | --- | --- |
|  |  | RV End-Diastolic Volume | RV End-Systolic Volume | RV Stroke Volume | RV End-Systolic Pressure | RV End-Diastolic Pressure | LV End-Diastolic Volume | LV End-Systolic Volume | LV Stroke Volume | LV End-Systolic Pressure | LV End-Diastolic Pressure |
| **Parameters** | T_0,LV­_ (kPa) | 0.140 | 0.024 | 0.264 | 0.168 | -0.099 | -0.089 | -0.387 | 0.273 | 0.284 | -1.562 |
|  | T_0,SEPTUM­_ (kPa) | 0.024 | -0.003 | 0.053 | 0.034 | -0.041 | -0.015 | -0.073 | 0.055 | 0.050 | -0.263 |
|  | T_0,RV­_ (kPa) | -0.146 | -0.383 | 0.109 | 0.138 | -0.843 | 0.062 | 0.030 | 0.101 | 0.083 | 0.595 |
|  | C_LV_ (Pa) | -0.019 | -0.006 | -0.034 | -0.016 | 0.098 | -0.040 | -0.045 | -0.035 | -0.037 | 0.382 |
|  | C_SEPTUM_ (Pa) | -0.006 | -0.001 | -0.012 | -0.005 | -0.015 | -0.013 | -0.013 | -0.013 | -0.013 | 0.156 |
|  | C_RV_ (Pa) | -0.025 | -0.025 | -0.026 | -0.035 | 0.411 | -0.018 | -0.015 | -0.023 | -0.017 | -0.123 |
|  | C_sa_ (Pa) | 0.017 | -0.005 | 0.039 | -0.025 | -0.477 | -0.054 | -0.135 | 0.045 | -0.130 | -1.036 |
|  | C_sv_ (Pa) | -0.053 | -0.062 | -0.044 | -0.091 | -0.817 | -0.029 | -0.029 | -0.030 | -0.037 | -0.733 |
|  | C_pa_ (Pa) | -0.080 | -0.167 | 0.014 | -0.240 | -0.723 | 0.011 | 0.013 | 0.009 | -0.002 | -0.132 |
|  | C_pv_ (Pa) | -0.025 | -0.037 | -0.013 | -0.053 | -0.483 | -0.020 | -0.021 | -0.020 | -0.026 | -0.593 |
|  | R_sa_ (kPa*ms/mL) | -0.231 | -0.179 | -0.286 | -0.203 | -0.750 | 0.089 | 0.409 | -0.300 | 0.393 | 1.599 |
|  | R_pa_ (kPa*ms/mL) | 0.136 | 0.343 | -0.087 | 0.419 | 0.643 | -0.086 | -0.091 | -0.080 | -0.069 | -1.008 |
|  | V_sv,0_ (mL) | -0.399 | -0.435 | -0.360 | -0.675 | -2.287 | -0.300 | -0.284 | -0.321 | -0.357 | -2.367 |
|  | V_pv,0_ (mL) | -0.222 | -0.263 | -0.178 | -0.404 | -1.614 | -0.198 | -0.198 | -0.199 | -0.242 | -1.749 |
|  | V_sa,0_ (mL) | -0.328 | -0.368 | -0.286 | -0.568 | -2.045 | -0.262 | -0.252 | -0.275 | -0.313 | -2.148 |
|  | V_pa,0_ (mL) | -0.194 | -0.229 | -0.156 | -0.349 | -1.407 | -0.161 | -0.159 | -0.164 | -0.195 | -1.504 |

# Bibliography

Alastruey, J., Xiao, N., Fok, H., Schaeffter, T., & Figueroa, C. A. (2016). On the impact of modelling assumptions in multi-scale , subject-specific models of aortic haemodynamics. *Journal of The Royal Society Interface*, *13*(119). https://doi.org/http://dx.doi.org/10.1098/rsif.2016.0073

Cuomo, F., Ferruzzi, J., Agarwal, P., Li, C., Zhuang, Z. W., Humphrey, J. D., & Alberto Figueroa, C. (2019). Sex-dependent differences in central artery haemodynamics in normal and fibulin-5 deficient mice: Implications for ageing. *Proceedings of the Royal Society A: Mathematical, Physical and Engineering Sciences*, *475*(2221). https://doi.org/10.1098/rspa.2018.0076

Evans, A. J., Iwai, F., Grist, T. A., Sostman, H. D., Hedlund, L. W., Spritzer, C. E., Negro-Vilar, R., Beam, C. A., & Pelc, N. J. (1993). Magnetic resonance imaging of blood flow with a phase subtraction technique. In vitro and in vivo validation. *Investigative Radiology*, *28*(2), 109–115. https://doi.org/10.1097/00004424-199302000-00004

Janssen, B. J. A., de Celle, T., Debets, J. J. M., Brouns, A. E., Callahan, M. F., & Smith, T. L. (2004). Effects of anesthetics on systemic hemodynamics in mice. *American Journal of Physiology - Heart and Circulatory Physiology*, *287*(4 56-4), 1618–1624. https://doi.org/10.1152/ajpheart.01192.2003

Pauca, A. L., O’Rourke, M. F., & Kon, N. D. (2001). Prospective evaluation of a method for estimating ascending aortic pressure from the radial artery pressure waveform. *Hypertension*, *38*(4), 932–937. https://doi.org/10.1161/hy1001.096106
